# Advancing Central Nervous System Drug Delivery with Microtubule-Dependent Transcytosis of Novel Aqueous Compounds

**DOI:** 10.34133/bmr.0051

**Published:** 2024-07-24

**Authors:** Mingzhu Zhang, Shaoqi Zhong, Lujing An, Pan Xiang, Na Hu, Wei Huang, Yupeng Tian, Giuseppe Battaglia, Xiaohe Tian, Min Wu

**Affiliations:** ^1^ Huaxi MR Research Centre (HMRRC), Functional and Molecular Imaging Key Laboratory of Sichuan Province, Department of Radiology and National Clinical Research Centre for Geriatrics, West China Hospital of Sichuan University, Chengdu, China.; ^2^ The Province Key Laboratory of the Biodiversity Study and Ecology Conservation in Southwest Anhui, School of Life Science, Anqing Normal University, Anqing 246011 China.; ^3^ Department of Chemistry, Key Laboratory of Functional Inorganic Material Chemistry of Anhui Province, Hefei 230039, China.; ^4^ West China Biobanks, Clinical Research Management Department, West China Hospital of Sichuan University, Chengdu 610000, China.; ^5^ Institute for the Physics for Living Systems and Department of Chemistry, University College London, London WC1H 0AJ, UK.; ^6^ Institute for Bioengineering of Catalunya (IBEC), The Barcelona Institute of Science and Technology, Barcelona, Spain.; ^7^ Catalan Institution for Research and Advanced Studies (ICREA), Barcelona, Spain.

## Abstract

The challenge of delivering therapeutics to the central nervous system due to the restrictive nature of the blood–brain barrier (BBB) is a substantial hurdle in neuropharmacology. Our research introduces a breakthrough approach using microtubule-dependent transcytosis facilitated by novel aqueous compounds. We synthesized a series of red-emitting pyran nitrile derivatives. The molecular structure of compounds, photophysical properties, and water solubility were characterized. BBB permeability of BN1 was assessed in an in vitro BBB model. The transmembrane transport mechanism was next analyzed. The derivative was injected in the wild-type mouse for evaluation of brain penetration and biodistribution in the brain. We further investigated the potential of BN1-functionalized BBB-nonpenetrated silica nanoparticles for brain targeting. This compound demonstrated an ability to form endosomes within the phospholipid layer, thus enabling efficient penetration of the BBB via microtubule-mediated transcytosis, as evidenced in vitro model. This was further confirmed by in vivo experiments that BN1 displays the excellent BBB penetration and retained in brain parenchyma. Furthermore, BBB-impermeable mesoporous silica nanoparticle codelivery system markedly enhanced the transport efficiency to the brain in vivo by BN1-functionalized. These findings indicate that our designed aqueous molecules not only are capable of traversing the BBB but also serve as a viable new strategy for central-nervous-system-targeted drug delivery.

## Introduction

The blood–brain barrier (BBB) serves as a vital checkpoint in neuropharmacology, safeguarding the central nervous system (CNS) from potentially harmful substances while also presenting a formidable barrier to therapeutic agents [1,2]. The CNS is effectively safeguarded by physiological barriers, specifically the BBB and the blood–cerebrospinal fluid barrier. The BBB’s selective permeability is governed by brain microvascular endothelial cells (BMECs), which exhibit a phenotype that is finely tuned to the CNS environment. BBB is impermeable to many molecules, with less than 2% of most drugs reaching the CNS, by creating continuous tight junctions and overexpressing several types of molecular efflux pumps [3–5]. This results in a barrier that is virtually impenetrable to most molecules, including a vast majority of pharmaceutical compounds [6].

It is well known that nanotechnology has led to the emergence of various types of nanomaterials that are being considered as promising carriers due to their unique advantages. Many approaches including functionalized nanocarriers were widely used to transport drugs or other molecules (such as nucleic acids, proteins, or imaging agents) across the BBB [7]. The types of nanoparticles (NPs) were categorized as inorganic-based nanocarriers, polymer-based, and biomimetic-based [8]. The further application of brain drug delivery is still limited by the large size of NPs, poor targeting efficacy, and difficulties in production [9,10]. Hence, the design of a noninvasive approach for the delivery of macromolecules or therapeutics to the brain has been still at the forefront of research [11,12].

In the past few decades, numerous therapeutic delivery strategies have been demonstrated to effectively transport drug molecules across the BBB [13]. Among these strategies, modification of drug molecules has exhibited promising potential [14]. Recently, among the available CNS drugs, small molecules constitute the majority of successful CNS therapeutics owing to their capacity to traverse the BBB via passive or carrier-mediated mechanisms [15]. Hydrophobic molecules with small molecular weight and suitable lipophilicity (log*P* = 2.0 to 4.0) can potentially penetrate BBB through simple diffusion. For instance, curcumin-like analogs CRANAD [16–19], BODIPY-based small molecule [20], and cyanine-like DANIR probes all exhibit high BBB permeability [21–23]. However, their entry mechanism at the subcellular level under both ex vivo and in vivo remains poorly understood. It may be a hydrophobic substance that is not easily administered intravenously. Hydrophilic substances can overcome this problem well. Although among several classes of substances capable of penetrating the BBB, the capillary permeability of small polarity molecules decreased by over 2 orders of magnitude when compared to other organs [24–26]. Nevertheless, small molecules preserve exclusive advantages in CNS diseases, including ability to balance aqueous and lipid solubility and low molecular weight [27].

The ligand-modified NPs can respond to receptors and increase BBB permeability than NPs without modification. Among the various strategies to ferry drugs across the BBB, mesoporous silica NPs (MSNs) have emerged as a versatile platform due to their biocompatibility, tunable pore size, and surface modification capabilities [28,29]. When conjugated with our novel compounds, these MSNs are designed to leverage the active transport mechanisms of the BBB [30]. The compounds act as targeting agents that enhance the MSNs’ ability to navigate the complex vascular landscape of the CNS, enabling them to cross the BBB efficiently [31].

In our study, we specifically focus on the design and application of 3 red-emissive, 2-photon excitable dicyanyle derivatives (BN, BN1, and BN2) for active CNS targeting. The water-soluble derivative BN1, in particular, demonstrates a rapid internalization with brain BMECs and effective penetration of a 3-dimensional (3D) BBB cell model. This is primarily facilitated by microtubule-mediated active transcytosis, a mechanism that we found to be substantially enhanced by the presence of BN1-functionalized MSNs. These findings are pivotal, as they illustrate a synergistic effect where the MSNs, aided by the BN1 compound, can cross the BBB and deliver therapeutic agents directly to the CNS.

Our in vivo studies further reinforce the potential of this approach, with BN1-functionalized MSNs showing the ability to cross brain capillaries and localize within multiple brain regions. This pioneering work not only demonstrates the active transport capabilities of hydrophilic small molecules but also underscores the role of MSNs as a critical vehicle in CNS drug delivery. We provide the first evidence that a combination of hydrophilic small molecules and MSNs can be effectively used for targeted therapeutic delivery across the BBB.

## Materials and Methods

### Materials and instruments

All chemicals and solvents were dried and purified by usual methods. Infrared (IR) spectra (4,000 to 400 cm^−1^), as KBr pellets, were recorded on a Nicolet FT-IR (Fourier transform IR) 170 SX spectrophotometer. Mass spectra were obtained on a Micromass GCT-MS spectrometer. ^1^H and ^13^C nuclear magnetic resonance (NMR) spectra were recorded on a Bruker AV 400 spectrometer. Ultraviolet (UV)-visible absorption spectra were performed by the UV-265 spectrophotometer. Fluorescence measurements were recorded on a Hitachi F-7000 fluorescence spectrophotometer. Before fluorescence experiments were performed, the fluorescence spectra were corrected by standard method. When the fluorescence measurements were taken, the concentration of samples was 1 × 10^−5^ M with quartz cuvette (path length = 1 cm). In the measurements of emission spectra, the slit width was 5 nm. The exciting voltage of emission spectrum was 400 V. For time-resolved fluorescence measurements, the fluorescence signals were collimated and focused onto the entrance slit of a monochromator with the output plane equipped with a photomultiplier tube (HORIBA HuoroMax-4P). The decays were analyzed by “least-squares”. The quality of the exponential fits was evaluated by the goodness of fit (χ^2^).

### Synthetic procedures

For the details of the BN, BN1, and BN2 synthetic procedures, see the Supplementary Materials.

### Two-photon excited fluorescence spectroscopy and 2-photon absorption cross-section

Two-photon absorption (2PA) cross-sections of all ligands were obtained by the 2-photon excited fluorescence method with femtosecond laser piles and a Ti:sapphire system (680 to 1,080 nm, 80 MHz, 140 fs) as the light source. The 2-photon action cross-section *σФ* values were determined by the following equation: $\begin{array}{c} \Phi_{\Delta }^{\text{unk}}=\Phi_{\Delta }^{\text{ref}}\times (m^{\text{unk}}\times F^{\text{ref}})/(m^{\text{ref}}\times F^{\text{unk}}) \end{array}$

Here, the subscripted ref represents the reference molecule (typically Fluorescein). *Φ* stands for the quantum yield, *n* is the refractive index, *F* is the integral area under the corrected emission spectrum, *c* is the concentration of the solution (in moles per liter). The *σ*_ref_ value of reference was cited from the literature [32].

### MTT assay

The cytotoxicity of the BN1 toward adherent cells was studied by MTT assay. HeLa and bEnd.3 cells were detached with trypsin, seeded into 96-well plates (100 μl per well) at a density of 5 × 10^4^ cells/ml, and incubated in a humidified incubator at 37 °C for 24 h. Then, adherent HeLa and bEnd.3 cells were treated with increasing concentrations of the compound (2.5 × 10^−7^ – 1 × 10^−4^ M) in the growth medium at 37 °C in 96-well plates. After 24 h of incubation, MTT (5 mg/ml, 10 μl) was added to each well and incubated for additional 4 h. The supernatant was then removed, and 100 μl of dimethyl sulfoxide (DMSO) was added to dissolve the formazan crystals. In addition, the cell culture plate was shaken for 10 min until no particulate matter was visible. Absorbance in each well was measured at 570 nm using a microplate reader (BioTek, USA). The cell viability (%) was calculated according to the following equation: cell viability % = *A*/*B* × 100, where *A* represents the optical density of the wells treated with various concentrations of the compounds and *B* represents that of the wells treated with medium.

### In vitro 3D cell culture and assessment of barrier properties

For mouse brain endothelial cells [bEnd.3, American Type Culture Collection (ATCC), CRL-2299], the medium used was Dulbecco’s modified Eagle’s medium (DMEM) supplemented with 10% fetal bovine serum, penicillin, streptomycin, l-glutamine, and Fungizone. Astrocyte (ATCC, CRL-2541, C8-D1A astrocyte type I clone) medium was antibiotic-free DMEM supplemented with 10% fetal bovine serum and l-glutamine. For transwell experiments, both sides of the transwell insert filters (Corning 3460 PE filter, diameter: 1.05 cm and pore size: 0.4 μm) were precoated with collagen (10 μg/cm^2^) for 2 h at room temperature. This was followed by seeding bEnd.3 endothelial cells on the upper surface of the transwell at a density of 2 × 10^4^ to 4 × 10^4^ cells per well and incubated for 12 h at 37 °C in 95% air and 5% CO_2_ to allow the cells to fully attach. Next, the astrocytes (1 × 10^4^ to 2 × 10^4^ cells per well) were seeded on the bottom of the transwell plate and incubated for 7 d at 37 °C, and the medium was being changed every 2 d. Light phase imaging was used for morphological observation of the BBB model by phase contrast optical microscopy. Tight junction formation and protein expression were characterized for both the 2D and 3D models with immunofluorescence assay [33]. Transendothelial electrical resistance (TEER) was recorded for all in vitro BBB models as a measure of junctional tightness. The relative resistance value was recorded to calculate the TEER values as follows: TEER = (*R*_t_ − *R*_b_) × *A*, where *R*_t_ and *R*_b_ represent the total resistance and background resistance, respectively, and *A* is the transwell area. The TEER value is directly correlated with the permeability of BBB for transport of extracellular molecule [34].

### Confocal microscopy of transwell filters

Compound BN1 were added at a concentration of 2.5 μM into the apical (upper) transwell compartment after TEER measurements were taken with an EVOM2 epithelial voltohmmeter. For the initial uptake experiments, cells were incubated for 0.5 to 2 h at 37 °C in 95% air and 5% CO_2_, the cell media of lower compartments were collected and recorded on a Hitachi F-7000 fluorescence spectrophotometer. The transwell insert membrane was excised using a scalpel and mounted on glass cover slip with VECTASHIELD mounting medium. Cells were imaged on Leica SP8 confocal laser scanning microscope with 63× oil immersion lens. For BN1, an excitation energy of 520 nm was used, and fluorescence emission was measured at 650 ± 20 nm. Nuclear staining was performed using 4′,6-diamidino-2-phenylindole (DAPI) (500 nM) for 10 min in phosphate-buffered saline (PBS). Image data acquisition and processing were performed using ImageJ.

### Cell imaging

Cells were cultured in 25-cm^2^ culture flasks in DMEM, supplemented with fetal bovine serum (10%), penicillin (100 U/ml), and streptomycin (50 U/ml) at 37 °C in a CO_2_ incubator (95% relative humidity and 5% CO_2_). Cells were seeded in 35-mm glass bottom cell culture dishes at a density of 1 × 10^5^ cells and were allowed to grow when the cells reached more than 60% confluence. For better cell imaging results, live cells were incubated with the complexes (10% water: 90% cell media) BN1 (excitation, 520 nm; emission, 650 ± 20 nm) at concentrations of 2.5 μM and maintained at 37 °C in an atmosphere of 5% CO_2_ and 95% air for incubation times ranging for 0.5 h. The cells were then washed with PBS (3 × 1 ml per well), and 1 ml of PBS was added to each well. The cells were imaged using Leica SP8 CLSM and oil immersion lenses. For colocalization, the bEnd.3 cells were incubated with CellMask Deep Red (excitation, 633 nm; emission, 641 ± 20 nm) or DAPI (excitation, 405 nm; emission, 450 nm) for 10 min after washing away the excess tracker by PBS 3 times. Two-photon confocal microscopy imaging of the compounds that were excited at 960 nm was acquired with a Carl Zeiss LSM 710, while the emission signals were detected in the region of 630 to 670 nm. For the cellular uptake assay, metabolic and endocytic inhibitors were used to treat the cells and check the uptake mechanism of BN1. Then, the cell media of lower compartments were collected and recorded on a Hitachi F-7000 fluorescence spectrophotometer. Image data acquisition and processing were performed using ImageJ.

### Reverse transcription polymerase chain reaction analysis

The mRNA expression levels of SLC22A2 (solute carrier protein 22A2), SLC22A5, and SLC47A1 were determined by reverse transcription polymerase chain reaction (RT-PCR). After the treatment with BN1 (0, 1, and 10 μM) for 1 h, total RNA from HepG2 cells were extracted using RNA extraction kit (Thermo Fisher Scientific), and RNA was reverse-transcribed to cDNA using the RevertAid First Strand cDNA Synthesis kit (Thermo Fisher Scientific) according to the manufacturer’s instructions. RT-PCR analysis was performed using the FastStart Universal SYBR Green master (Rox) (Roche) on the ABI StepOnePlus software. Primer pairs (Table S1) were designed and synthesized by Wuhan Biotechnology Co. Ltd. (Wuhan, China). The levels of transcripts were normalized using β-actin as an internal standard. Fold changes were calculated using the 2^−ΔΔ*C*t^ method. Levels of each different mRNA in the control cells were designated as 1, and the relative levels of the gene transcripts of the samples were expressed as the fold change. Analysis was carried out in triplicates.

### Gene knockout analysis

A lentiviral short hairpin RNA (shRNA) vector (Fig. S1) targeting SLC22A2, SLC22A5, and SLC47A1 was generated by inserting stranded oligonucleotides [shSLC22A2-1, 5′-ccggCTCTCAGCAAGTACGAGTTTGCTCGAGCAAACTCGTACTTGCTGAGAGTTTTTG-3′ (forward); shSLC22A2-2, 5′- ccggGGACAACATCTACTTGGATTTCTCGAGAAATCCAAGTAGATGTTGTCCTTTTTG-3′ (forward); shSLC22A5-1, 5′-ccggGATCGCTTCCTGCCTTATATTCTCGAGAATATAAGGCAGGAAGCGATCTTTTTG-3′ (forward); shSLC22A5-2, 5′-ccggGGACCTGGTGTGTAAGGATGACTCGAGTCATCCTTACACACCAGGTCCTTTTTG-3′ (forward); shSLC47A1-1, 5′- ccggGCTCAAGTACATGCCAATTTGCTCGAGGGAAATCAAGTTTGCCAGTGCTTTTTG-3′ (forward); shSLC47A1-2, 5′- ccggGCCATGGAGAAATTATGATGACTCGAGTCATCATAATTTCTCCATGGCTTTTTG-3′ (forward)] into pCDH-U6-GFP-EF1-Puro vector. bEnd.3 cells were infected with the shRNA vectors and selected with puromycin (1.25 μg/ml).

### In vivo near-IR imaging of BN1 penetration into the brain

Three-month-old male Kunming mouse with scalp hair removed were intravenously injected via the tail vein with BN1 (1 mg/kg, 20% DMSO, 80% propylene glycol, 100 μl). Fluorescence signals from the brain were recorded at different time points after tail vein injection of BN1 with an IVIS Spectrum Imaging System (Berthold, LB983). For the measurement, a filter set (excitation, 520 nm; emission, 650 nm) was used, and optical images were acquired using an exposure time of 1 s. During the imaging process, the mice were kept on the imaging stage with 2.5% isoflurane gas in an oxygen flow (1.5 l/min). Imaging data were analyzed by Living Image software, and a region of interest was drawn around the brain region.

### Preparation of BN1-loaded NPs and physicochemical characterization

MSNs were prepared by the previously reported method [35]. Briefly, CTAB (2.0 g) was added into the mixed solution of ethanol (10 ml) and water (76 ml) under magnetic stirring at 80 °C for 30 min. Then, triethanolamine (1 ml) was added into the transparent solution and stirred continuously for another 1 h. After that, TEOS (3 ml) was added into the resulting solution dropwise and continuously stirred for 12 h. Subsequently, the MSNs were obtained through centrifugation under 12,000 rpm for 10 min. The obtained MSNs were calcined at 600 °C for 6 h to remove the CTAB. MSN (0.25g) was added into 3 ml of deionized water containing 0.5 g of BN1 and then stirred at 25 °C for 12 h. After that, the BN1-loaded MSN was centrifuged under 11,000 rpm for 10 min and washed 5 times with water to remove free BN1. The composition of the nanoplatform was further characterized by the FT-IR spectrum (FT-IR spectrometer: iS10, Nicolet Co., USA). In addition, MSN@BN1 presented overlap peaks of MSN and BN1, confirming the successful load of BN1 into MSN.

### Ex vivo 2-photon microscopy imaging of mouse brain stained with dyes

Three-month-old male Kunming mouse were intravenously injected via the tail vein with BN1 (0.4 mg/kg, 200 μl). The compound solution was allowed to circulate for 5 min, following which the mice were terminally anesthetized and transcardially perfused with 0.1 M PBS (pH 7.4), followed by 4% (w/v) of paraformaldehyde in PBS. The perfused brains were extracted, dura-mater-removed, and then postfixed for 7 h in 4% paraformaldehyde at 4 °C. The fixed brains were immersed in 30% (w/v) of sucrose in PBS overnight at 4 °C for cryoprotection. Fixed brains were cut using a cryostat (Leica, 1950) at 20 μm in the coronal plane and mounted on glass slides. For microvasculature-stained, fluorescein-labeled lectins (FL-1174, Vector Labs, UK) (1:200) were incubated with brain slices at 37 °C for 1 h. For astrocyte and neuronal immunostaining, sections were initially washed 3 times for 5 min in PBS at room temperature and preincubated for 1 h in antibody-blocking buffer consisting of 2% goat serum and 1% bovine serum albumin in 0.1% PBS-Triton X-100. After washing with PBS, processed sections were incubated with primary antibodies diluted in PBS for 1 h at room temperature: polyclonal rabbit anti-glial fibrillary acidic protein (GFAP) (Abcam; 1:500), rabbit anti-neuron-specific nuclear protein (NeuN) (Abcam; 1:500), rabbit anti-CD146 (Abcam; 1:250), and rabbit anti-microtubule-associated protein 2 (MAP2) (Abcam; 1:500). Sections were washed 4 times in PBS for 5 min each. Sections were then incubated for 2 h at room temperature using the following secondary antibodies: goat anti-rabbit Alexa Fluor 488 (1:200) produced by Molecular Probes (Abcam). After intensive washing in PBS, sections were mounted on glass slides, the nuclei were stained with DAPI (500 nM in PBS) for 1 min and coverslipped using an aqueous mounting medium (VECTASHIELD, Vector Labs). Slides were then imaged using laser scanning confocal microscopy (Zeiss, LSM 510) for emission of GFAP, MAP2, and NeuN (excitation, 488 nm; emission, 500 to 550 nm). Image data were acquired and processed using ImageJ software.

### Animals

All procedures involving animals were approved by and conformed to the guidelines of the Institutional Animal Care Committee of The West China Hospital of Sichuan University. We have taken great efforts to reduce the number of animals used in these studies and taken effort to reduce animal suffering from pain and discomfort.

### Statistical analyses

Statistical analyses were carried out with GraphPad Prism6 software, and data were presented as means ± SD. Statistical comparisons between multiple groups were performed using one-way analysis of variance (ANOVA), followed by Student’s *t* test. *P* < 0.001 was considered statistically significant.

## Results

### Synthesis and characterizations

**Fig. 1.**
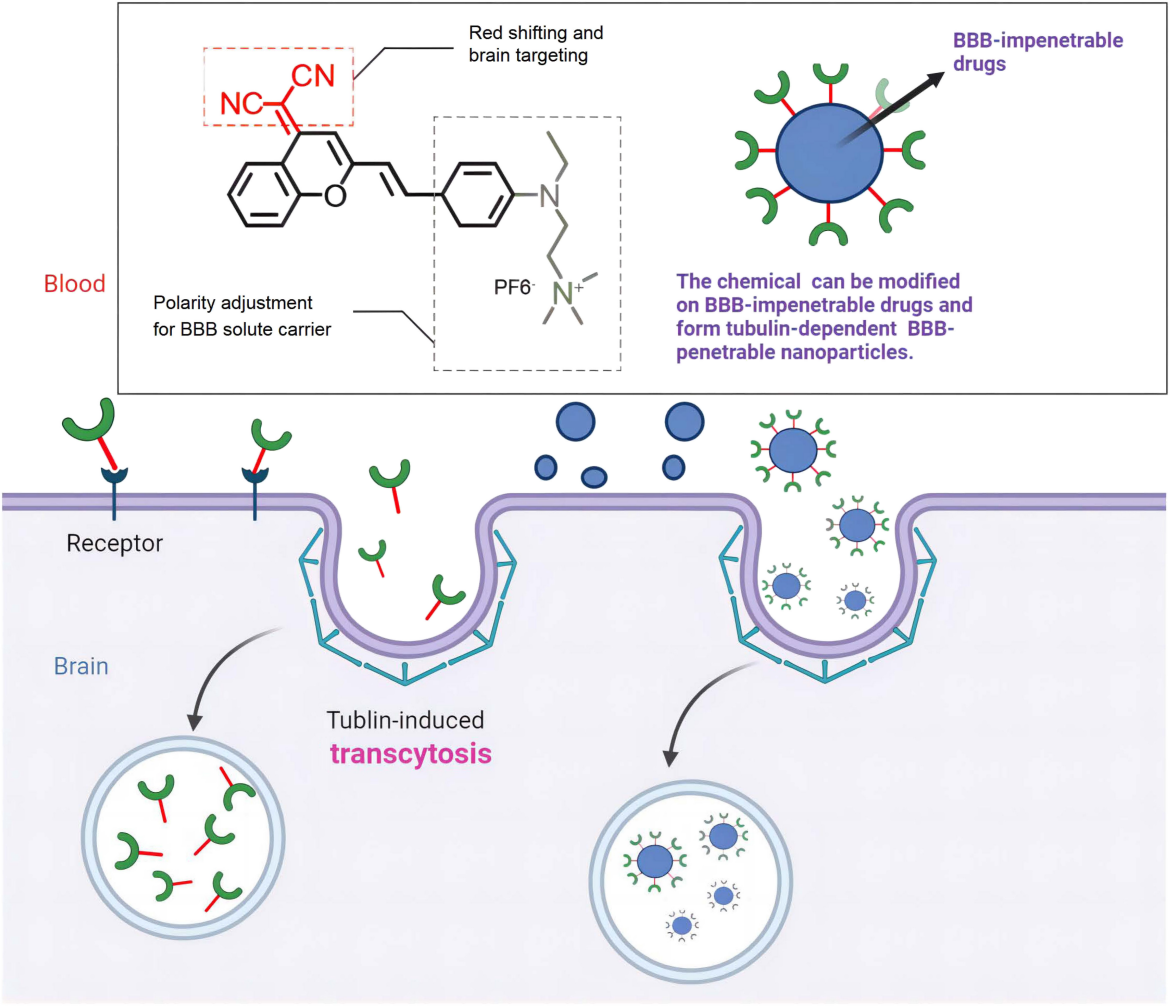
Schematic representation for transportation across the BBB and the proposed compound design strategies for BBB penetration.

The 3 pyran nitrile derivatives (BN, BN1, and BN2) (Fig. S2) was constructed with the following considerations: (a) These derivatives are linked 2-benzopyran dinitrile group with the *N*,*N*-diethylation to red-shift the absorption byalkenyl and phenyl group π-bridge [21]. (b) The quaternary ammonium salt groups (BN1 and BN2) were added at the diethylamino terminal, compared to previously reported BBB-penetrating molecules (Fig. 1); their polarity and positive charge affinity with phospholipid layer were substantially enhanced. What’s more, on the basis of the symmetric charge transfer, different electron donor capacities of terminal groups could enhance the values of 2PA cross-sections (δ) [36]. (c) Evidence suggests that the dicyanyl group could facilitate BBB penetration for brain targeting [18], while its actual molecular mechanism remained unclosed.

Figure S2 gives the structures of the 3 molecules based on the above regard. After purification, the molecular structure of compounds was confirmed by ^1^H NMR, ^13^C NMR, mass spectra (Figs. S3 to S8), FT-IR, and elemental analysis. All the detailed synthetic procedures and characterizations were given in the Supplementary Materials. Detailed methods and approaches were listed in the Supplementary Materials.

### Photophysical properties

**Fig. 2.**
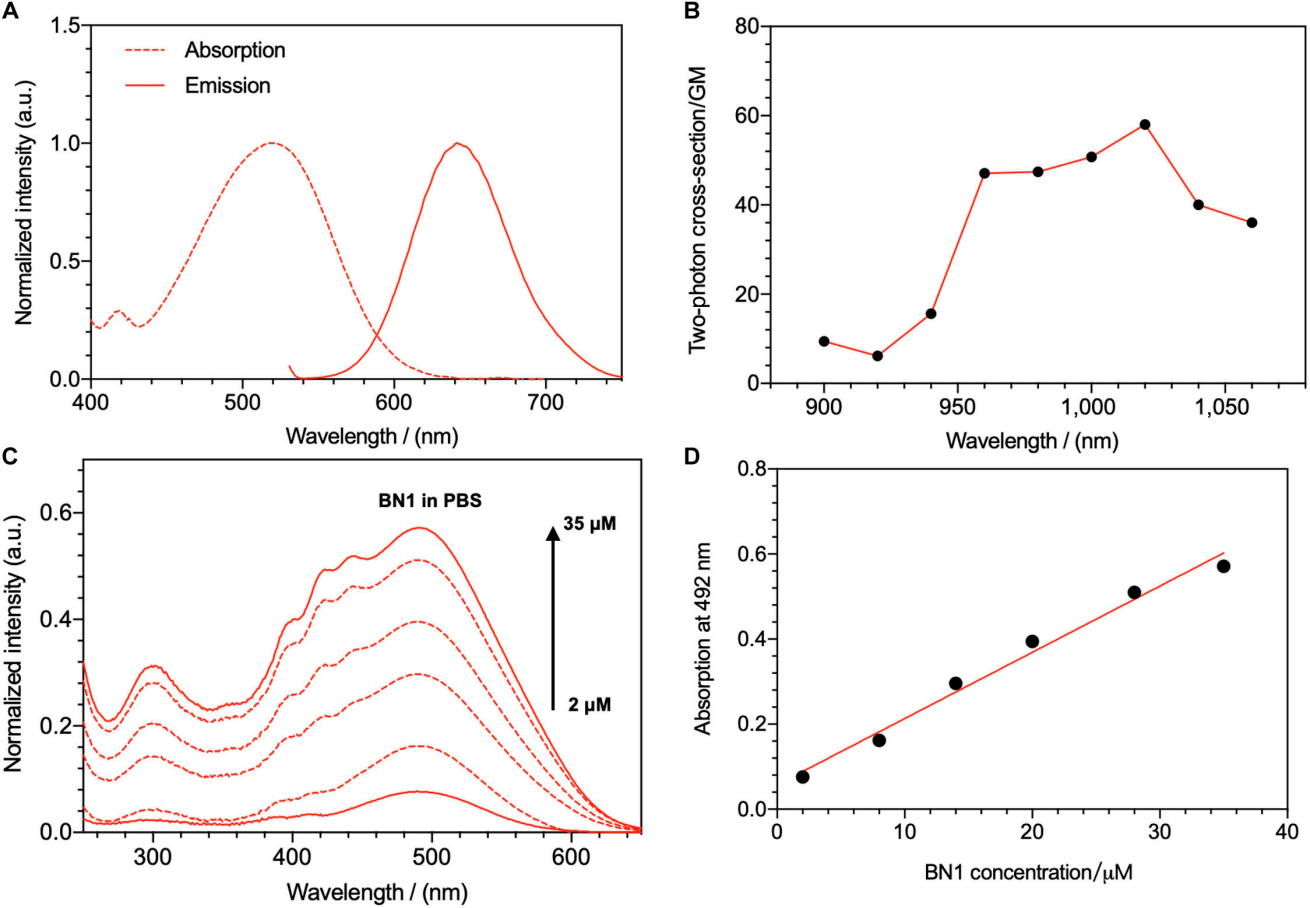
(A) UV-visible absorption and fluorescence emission spectra of BN1 in 10 μΜ PBS solution. (B) 2PA cross-sections BN1 (50 mM) in DMSO solutions from 900 to 1,060 nm. (C) Absorption spectra of BN1 in H_2_O buffered with PBS. (D) Plot of intensity against the BN1 concentration. a.u., arbitrary units.

The maximum absorption bands and the fluorescence spectra of the compounds (Fig. 2A and Fig. S9) exhibited a blueshift in the order of BN2 < BN1 < BN with the increasing electron-donating ability of terminal aromatic amino moiety. According to time-dependent density functional theory calculations (Table S2 and Fig. S10), the lower energy band of BN1 around 520 nm was assigned to the intramolecular charge transfer from the benzopyran dinitrile to quaternary ammonium groups. BN to BN2 all exhibited large Stokes shift (>100 nm), which can eliminate the influence of excitation spectra in cell imaging. While the 2PA cross-section (Fig. 2B and Fig. S11) exhibited the sequence of BN1 > BN2 > BN at 1,020 ± 20 nm, the results that BN1 performed the highest 2PA cross-section should stem from the increased extent of symmetrical charge transfer within molecule [36].

Compared with BN, the electron-donating capacity of the aromatic amino group within BN1 is weakened, which results in the enhanced transition dipole moment and 2-photon activity. In addition, comparing the other derivatives, BN1 has the highest quantum yield (24.2%) and moderate fluorescence lifetime (Fig. S12). The linear absorption behavior confirmed that BN1 possessed good water solubility in a concentration range of 2 to 35 μM (Fig. 2C and D).

### BBB permeability in an in vitro BBB model

To evaluate the cellular uptake of the 3 compounds, HeLa cells (cancerous cervical cells) and bEnd.3 cells (mouse BMECs) were used (Fig. S13). All the compounds entered both cell types quickly with similar distribution, while the signal from the hydrophilic (log*P* = −1.54) molecule BN2 in both 2 cell types was considerably lower, indicating poorer cellular uptake [37]. Cell treated with BN1 displayed intense luminescence with HeLa cells having the most intense signal from the cytoplasm, while the bEnd.3 cells from their membrane (Figs. S14 and S15) in 2D BBB model. MTT assay for BN1 showed low toxicity toward both HeLa and bEnd.3 cells (Fig. S16), and, thus, we infer that the entry is not associated with membrane disruption.

**Fig. 3.**
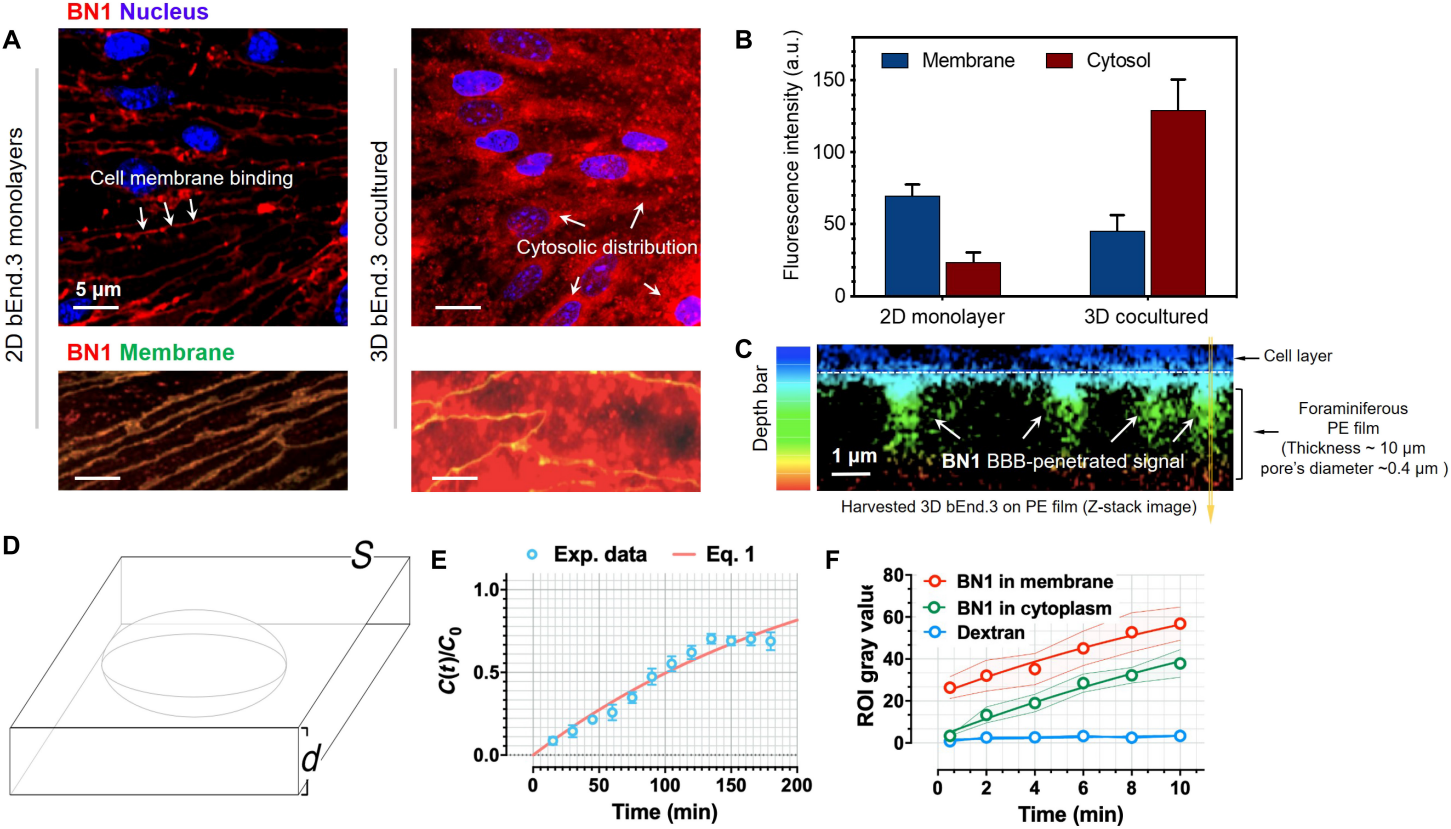
(A) Schematic representation of the in vitro 2D and 3D models of BBB. 2D bEnd.3 monolayers on glass-bottom dishes; 3D bEnd.3 cocultured with astrocytes on transwell insert treated with BN1 and costained with DAPI. (B) Relative fluorescence intensity analysis of cellular uptake BN1 in membrane and cytosol in 2D and 3D BBB models. (C) Depth code of 3D Z-stack transwell insert microporous membrane after treating with BN1. PE, phycoerythrin. (D) Kinetic model to describe BN1 transport across the BBB model. (E) Transwell permeability assay with fitting Eq. 1, time course of fluorescence from BN1 in cell media of lower compartments. (F) Region of interest gray value of BN1 in cell membrane (red line), cytoplasm (green line) in 3D model, and dextran–fluorescein isothiocyanate (blue line) in whole cell in the same time period.

We counterstained cells with CellMask, a marker for the plasma membrane (Fig. 3A, left), showing that BN1 colocalized with it. We further evaluated BN1 BBB permeability in a cocultured 3D BBB model, using a well-established transwell system [38]. The BBB integrity was proved by assessing the relevant tight junction protein expression and the TEER value (Fig. S17). BN1 live cellular uptake showed a different distribution in polarized (3D) bEnd.3 cells (Fig. 3A, right), whereas here is distributed across the whole cell opposite to localize at the membrane (Fig. 3B). Therefore, the 3D models showed transwell filter membrane fluorescence (Fig. 3C), confirming that the BN1 has BBB permeability.

Further, time-dependent assay demonstrated that BN1 was internalized through the endothelial cell layer efficiently (Fig. S18). 3D bEnd.3 monolayer images showed that BN1 penetrated the endothelial monolayer across (Fig. S19A and B), and the imaging results showed that BN1 was transferred from the entire cell membrane to the cell in the form of dot signals within 5 min. We derived a simple kinetic model (Fig. 3D) to describe BN1 transport across the brain endothelial cells, and this allowed us to estimate the ration between BN1 concentration inside the cells, *c*(*t*), and BN1 concentration in solution at time zero, *c_B_*, according to

$$\frac{c\left(t\right)}{c_{B}}=\frac{D}{\kappa d^{2}}\left(1-e^{-\kappa t}\right)$$ (1)

where *D* is the BN1 diffusion coefficient across the cell, *d* is the cell thickness, and $\kappa =\left(\frac{D}{d^{2}}-K\right)$ is the overall rate, with *K* = *k_in_* − *k_out_* being the net pumping rate. We assume that the fluorescence per cells that we measured as a function of time changes linearly with BN1 concentration, and we can thus assume $\frac{c\left(t\right)}{c_{B}}≅\frac{F_{C}\left(t\right)}{F_{B}\left(0\right)}$ and fit the experimental data using Eq. 1 (Fig. 3E), with similar increasing trend in membrane and cytosolic intensity (Fig. 3F), compared with a large molecule (dextran). We calculated the BN1 diffusion coefficient across the cells of about *D* = 10^−14^ m^2^/s and net pumping rate. *K* = *k_in_* − *k_out_* = −6 × 10^−6^ s^−1^, suggesting that process is dominated by active flux BN1. Moreover, the negative sign suggests that flow is directed from the cell out, i.e., *k_in_* < *k_out_*.

The transmembrane transport mechanism of hydrophilic molecules is always considered to be several highly specific SLCs expressed by BBB [39]. The chemical nature of BN1 suggests that its transport is controlled by SLC organic cation transporters well known to be up-regulated at the BBB [40]. We thus examined the expression of organic cation transporter-2 (OCT-2, SLC22A2), organic cation-sodium transporter-2 (OCTN-2, SLC22A5), and multidrug and toxic compound extrusion protein-1 (MATE1, SLC47A1) by RT-PCR. As shown in Fig. S20, the mRNA of OCT-2, OCTN-2, and MATE1 observably decreased after being exposed to BN1, indicating that it suppressed the transporters to cross the BBB. Furthermore, simultaneous knockdown of the 3 SLCs genes had a greater effect on increasing BN1 ingestion sensitivity than silencing them individually (Fig. S21). This may mean that the small cationic compound BN1 could be taken up by endothelial cells through a special pathway.

**Fig. 4.**
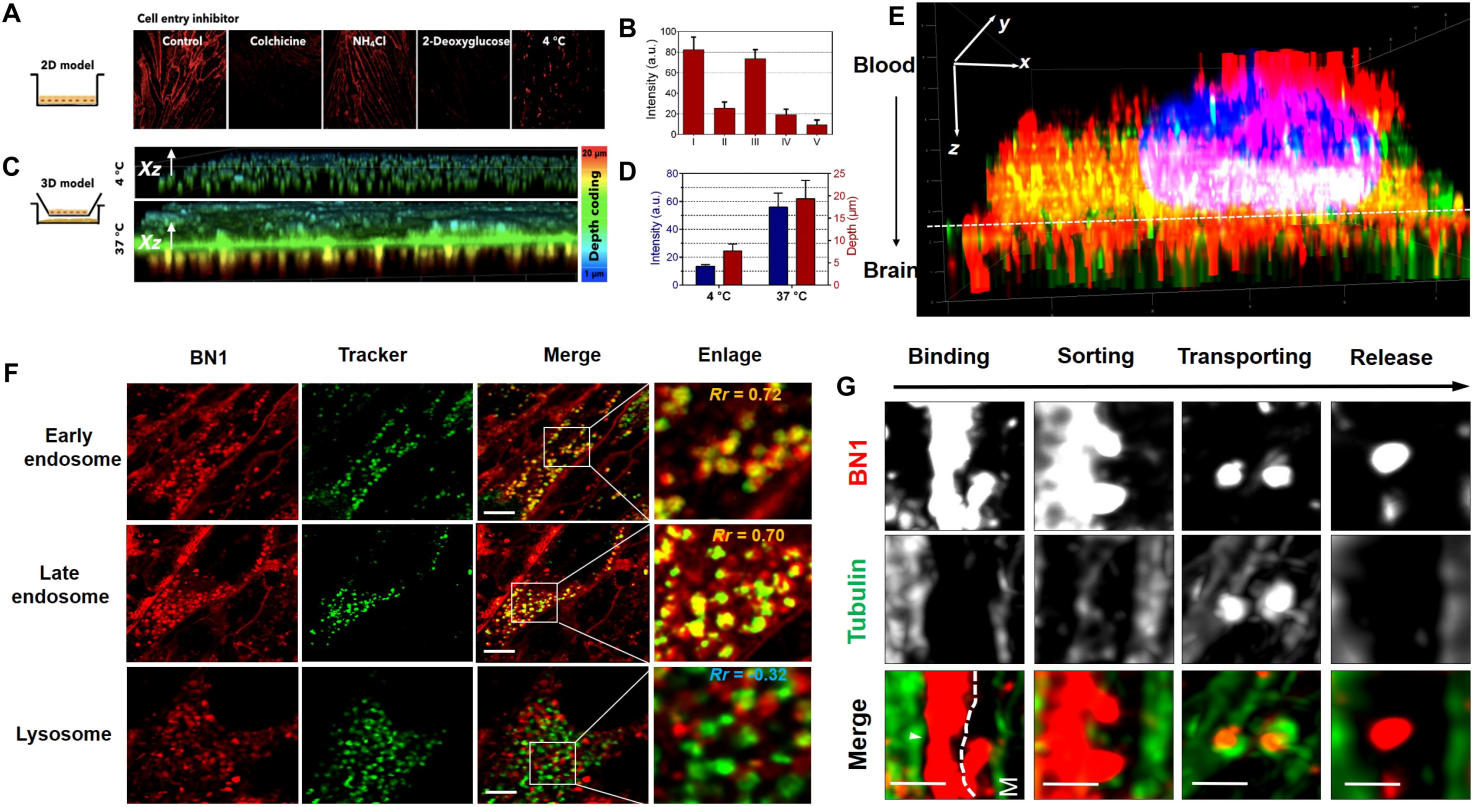
(A) Treatment with the different cell entry inhibitors: (I) control, (II) colchicine, (III) NH_4_Cl, (IV) 2-deoxy-d-glucose, and (V) 4°C. Scale bars, 20 μm. (B) Fluorescent intensity analysis for living 2D monolayer bEnd.3 cells treated with BN1 and different inhibitors. (C) Depth code of 3D Z-stack transwell insert microporous membrane and cells for different temperatures. (D) The depth values of cellular transport BN1 crossing transwell insert microporous membrane and relative luminescence intensity of BN1 in cell media of lower compartments from (C). (E) bEnd.3 monolayer on glass bottom dishes (collagen-coated) treated with BN1 and costained with early endosome, late endosome, and lysosome with high magnification of the white rectangular regions, respectively. (F) 3D imaging of bEnd.3 cells in 3D BBB model treated with BN1 for 1 h costained with Sir-tubulin. Scale bars, 20 μm. Green dashes show the transwell-insert membrane. (G) The magnified region shows the interaction details of BN1and tubulin for different time periods. M, membrane. Scale bars, 1 μm.

To understand how compound BN1 penetration BBB, its cellular uptake mechanisms were investigated by well-documented endocytosis and active transport inhibitors [38]. 2D BBB model (Fig. 4A and B) micrographs and slightly cellular uptaking (Fig. S22) observed no luminescence under 4 °C and 2-deoxy-d-glucose treatment, indicating that BN1 entering cells was by an energy-dependent pathway and proving that BN1 is not a membrane-permeable molecule. The 3D models (Fig. 4C and D and Fig. S23) confirm the energy-dependent pathways again. No influence of BN1 accumulation after treating with the active transport inhibitors NH_4_Cl suggested that the molecules enter cells via nonlysosomal routes. Furthermore, colchicine could influence the uptake of BN1. It means that microtubule-mediated membrane and endosomal trafficking could involve in BN1 uptake.

To further assess the specific endocytosis pathway of BN1, it is noteworthy that the fluorescent puncta corresponded to early/late endosomes (Fig. 4E, *Rr* = 0.72/0.70). This phenomenon explains that the internalization of BN1 was within endosomes for trafficking during transport rather than lysosomes for degradation (LysoTracker, *Rr* = −0.32). This result was in a good agreement with the ammonium chloride inhibitors. To test whether transport of BN1 crossing BBB was indeed associated with microtubules, we performed live-cell microtubule imaging labeled with SiR-tubulin and then investigated BN1 trafficking in 3D BBB models (Fig. 4F). Transwell filter membrane pore fluorescence revealed that BN1 was shuttled across the bEnd.3 cells in BBB models closely associated with microtubules. Moreover, according to the results as shown in Fig. 4G, where the microtubules were tracked for BN1 trafficking crossing BBB in time-dependent assays, the endosome initiated BN1 formation along lumen side, then moved, and finally released by microtubules after arriving brain side. Together, the above data suggest that BN1 entered the cells via energy-dependent reverse clumps across the membrane and then undergone transcytosis, which depends on the microtubule-mediated transportation.

### In vivo imaging of BN1 penetration into the wild-type mouse brain

**Fig. 5.**
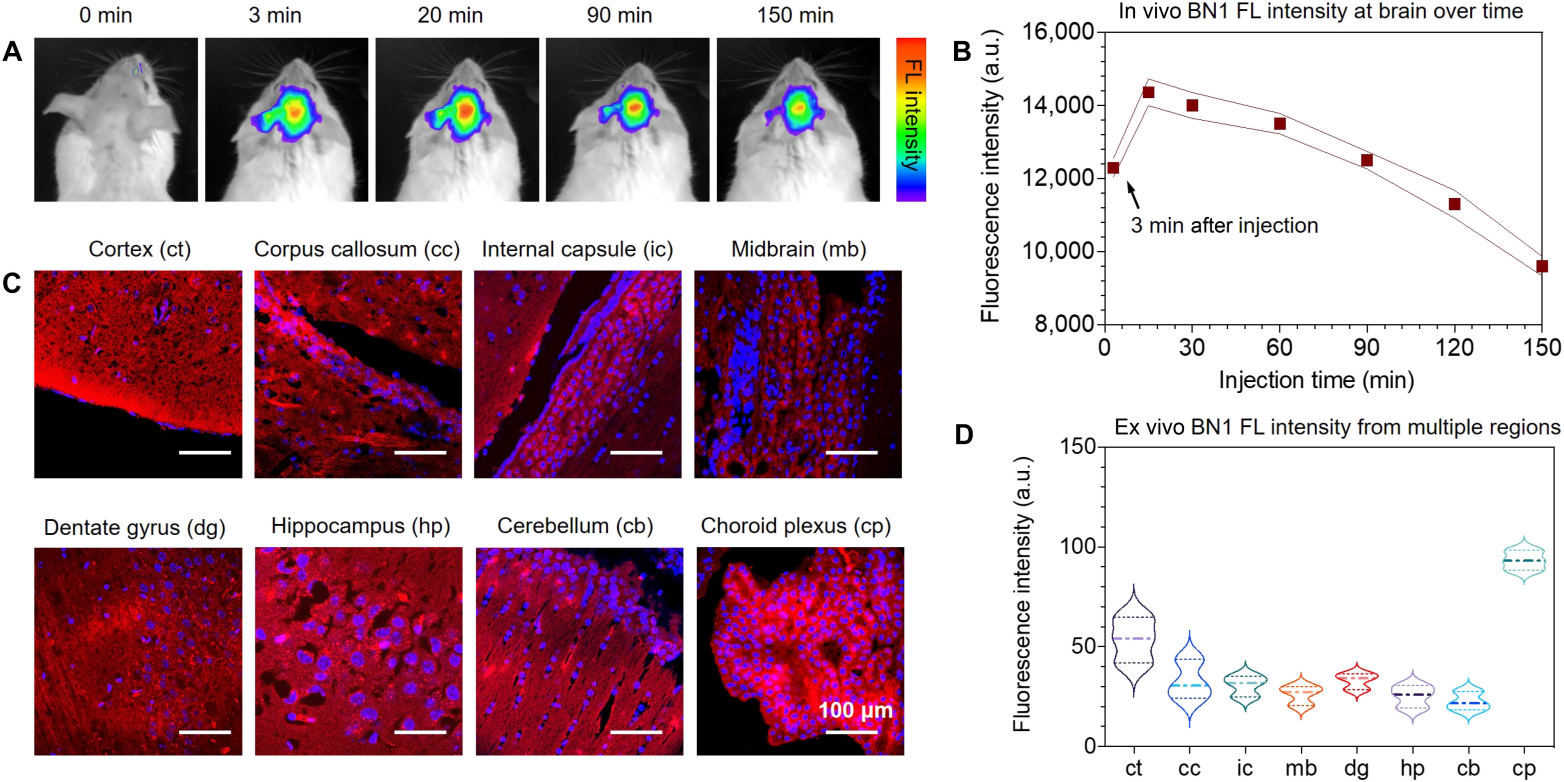
(A) Representative images of wild-type control mice at different time points before and after multiple intravenous (IV) injections with BN1 (1 mg/kg). (B) Quantitative analysis of fluorescence signals from mice (*n* = 3) at preinjection and 3, 20, 30, 60, 90,120, and 150 min after intravenous injection. (C) Confocal micrographs of different brain slices regions extracted from mice intravenous injection of BN1. (D) Relative luminescence intensity of BN1 in each region of (C). Scale bars, 100 μm.

To investigate the capability of BN1 to penetrate the BBB in vivo, we administered the molecule into mice and assessed the brain kinetics via the tail intravenous injection. BN1 could penetrate the BBB efficiently within 3 min (Fig. 5A). Brain kinetic curves were obtained by semiquantitative analysis of the images (Fig. 5B). The data follow a typical trend of the peripheral compartment of a 2-compartment pharmacokinetic model [41]. Fifty minutes after injection, its signal gradually decreased and remained at detectable levels even after 2 h. The deducted intensity might attribute to the specific binding BN1 in the brain and would provide an enhanced signal-to-noise ratio. It is not surprising that a relatively high signal of BN1 was detected in the kidney, especially close to the inner wall of the renal tubule (Fig. S24). To further confirm the tissue distribution of intravenously administered BN1, the frozen brains were cryosectioned. BN1 could be observed in all regions analyzed, including cortex, corpus callosum, internal capsule, midbrain, dentate gyrus, hippocampus, cerebellum, and choroid plexus (Fig. 5C and D). The results further confirmed that BN1 displays the excellent BBB penetration and retained in brain parenchyma.

### The biodistribution of BN1 in the brain after crossing the BBB

**Fig. 6.**
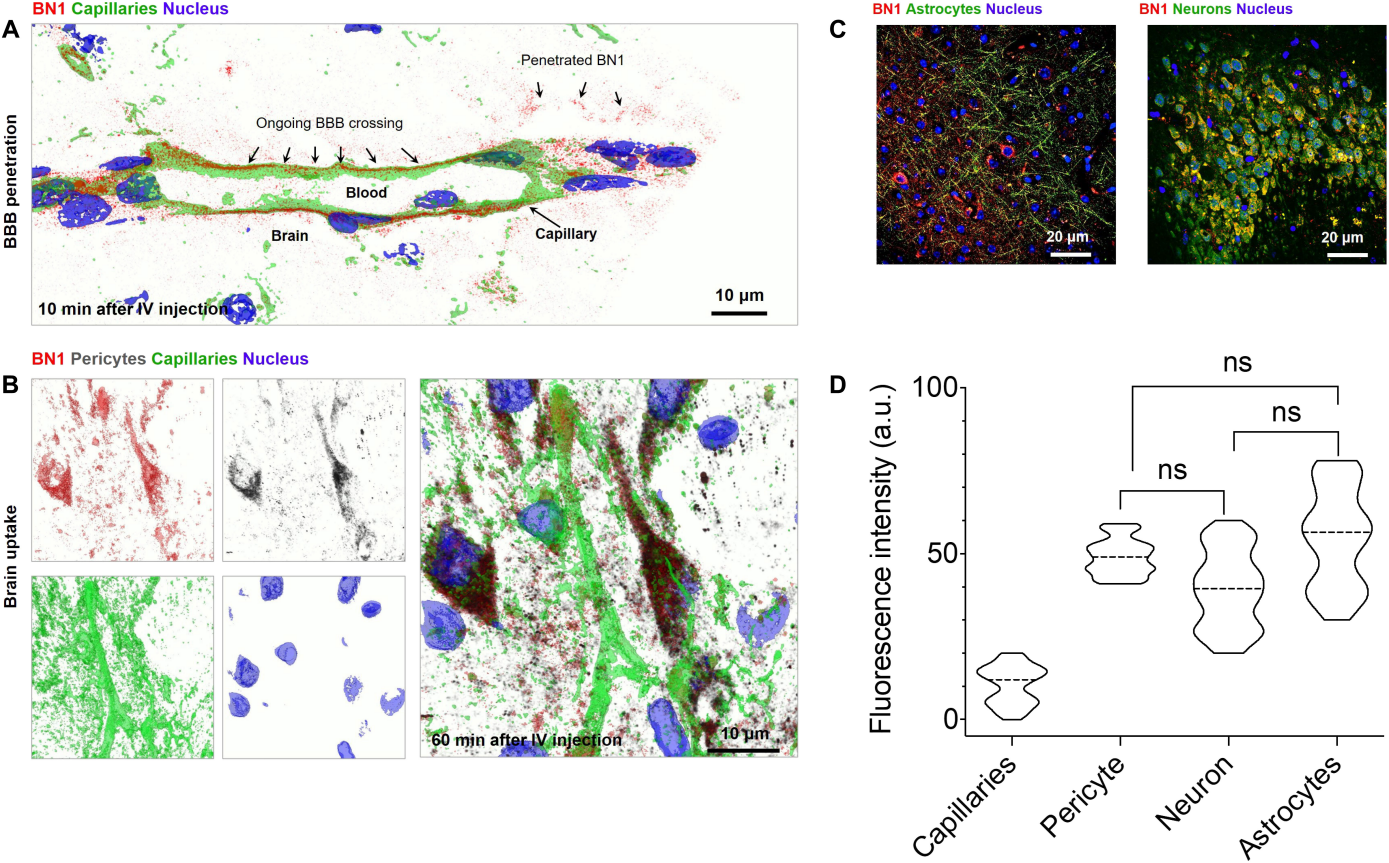
(A) Ex vivo assessment of BN1 (red) crossing in vivo BBB, colabeled with nucleus (blue) and capillaries (green); the enlarged micrographs of the white rectangular regions showed the detail of brain endothelium capillaries (green dashes) and BN1 (red) signal imaged by CLSM after intravenous injection of BN1. (B) Brain sections from mice after intravenous injection of BN1 (red) labeled Alexa Fluor 488-lectin to show brain capillaries and IF (immunofluorescence) assays to show pericytes (gray). (C) Confocal micrographs of brain sections from mice after intravenous injection of BN1 (red), IF assays to show neuronal end-feet (MAP2) astrocytes (GFAP) and neurons (NeuN), respectively. (D) Relative luminescence intensity of BN1 in different regions of after intravenous injection of BN1. Scale bars, 20 μm. ns, not significant.

Furthermore, BN1-treated mouse brain sections were evaluated at the signal cell level to assess which brain cells uptake the molecule. As shown in Fig. 6A, fluorescent lectin-labeled endothelium showed a high degree of BN1 that closely associated with the capillaries 30 min after injection. BN1 also can be found outside of the vessel in the brain parenchyma, indicating ongoing accumulation after BBB penetration. Further immunofluorescence experiments highlighted multiple cerebral cell types, again confirming that BN1 was indeed crossing the BBB and entering the brain parenchyma. As shown in Fig. 6B, administration of BN1 (red signal) was distributed on the brain endothelial and firstly entered capillary-wrapped pericytes (gray signal). Further analyses confirmed that BN1 enters pericytes (CD146), astrocytes (GFAP), and neurons (NeuN) as well as MAP2-positive cells (Fig. 6C and Fig. S25). Moreover, the average intracellular intensity analysis (Fig. 6D) from multiple representative micrographs suggested that capillary endothelial and pericytes indeed uptake more BN1 substances.

### Transporting BBB-nonpenetrated MSNs into the CNS using BN1

**Fig. 7.**
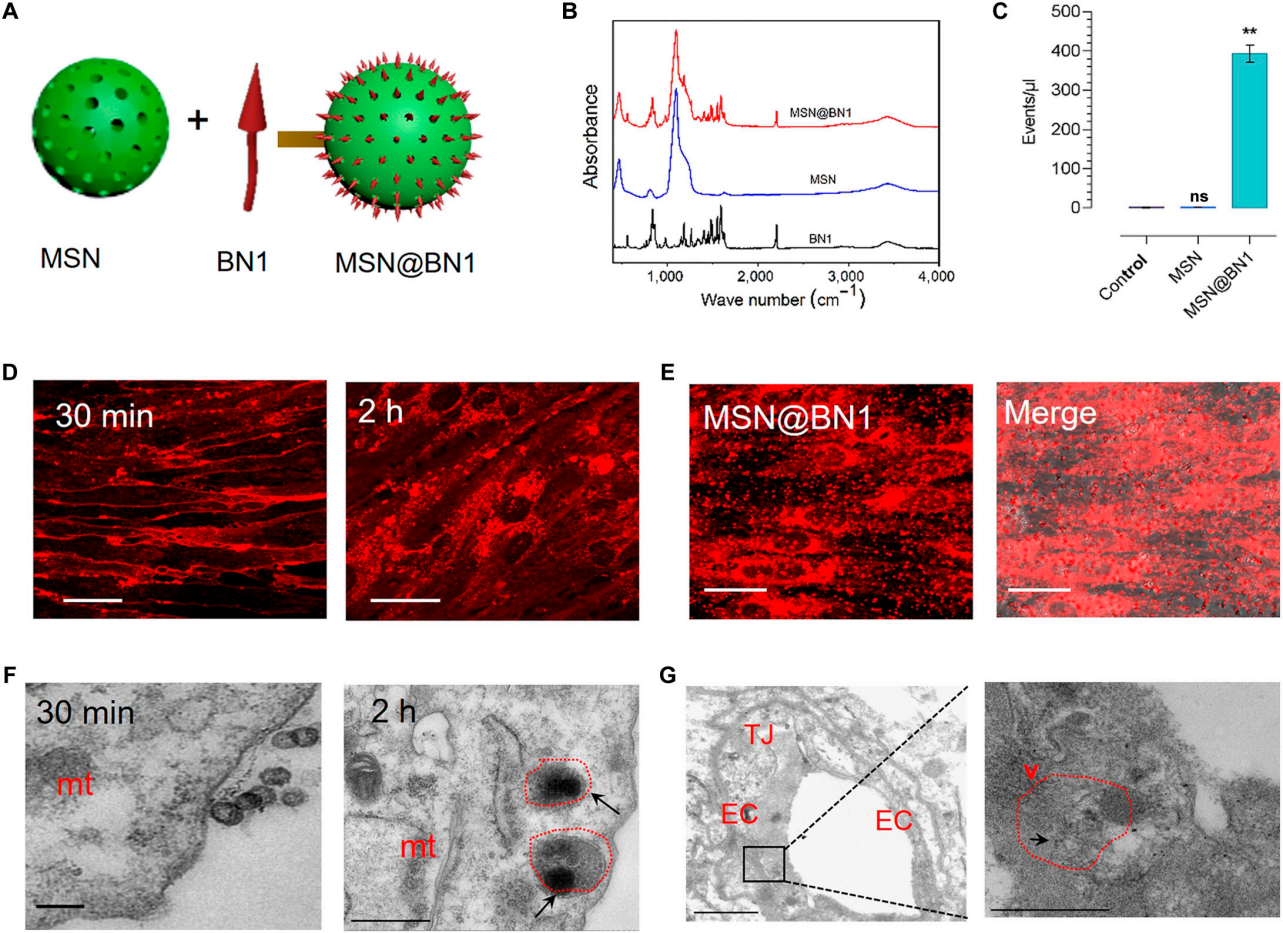
(A) Synthetic procedure of MSN@BN1. (B) FT-IR spectra of MSN@BN1 and BN1. (C) Flow cytometry intensity assay for cellular uptake of different samples. The data are expressed as the means ± SEM (*n* = 3). **P < 0.001 (D) 2D bEnd.3 monolayer on glass-bottom dishes treated with MSN@BN1 for different times. Scale bars, 20 μm. (E) 3D BBB model treated with MSN@BN1 for 30 min. Scale bars, 10 μm. (F) TEM images of 2D bEnd.3 treated with MSN@BN1 for different times. (G) TEM micrograph shows the brain microvascular section and (right) the higher resolution micrographs showing the details of ex vivo BBB crossing of MSN@BN1. Scale bars, 500 nm. mt, mitochondria; TJ, tight junction; EC, endothelial cells; mvb, multivesicular body; v, vesicles.

To further confirm whether BN1 with BBB permeability capable of acting as a vector for accelerating delivery of large molecules into the CNS, we next investigated the potential of BN1-functionalized silica NPs for brain targeting. Here, silica NPs (SiO_2_-NPs) were applied; because of their unique physicochemical properties, exceptionally large surface area and high drug-loading efficiency have been extensively exploited in biomedical fields. Several recent studies focused on the development of SiO_2_-NPs targeted and controlled drug delivery and release [42,43]. However, the SiO_2_-NPs as a candidate for drug delivery systems crossing BBB are rarely existed [44]. As shown in Fig. 7A, BN1 was successfully functionalized to the mesoporous SiO_2_-NPs (MSNs). Particle size analysis showed that the particle size of MSN and MSN@BN1 was approximately between 150 and 200 nm (Fig. S26). Then, the success of obtained MSN@BN1 hybrid was confirmed by IR spectroscopy (Fig. 7B). The parallel luminescence quantitative assay of MSN@BN1-treated live-brain endothelial cells carried out by a fluorescence activated cell sorter (Fig. 7C) was much brighter than that in free MSN control group, indicating the excellent cytocompatibility and aggregation of MSN@BN1 in BBB 2D model. In addition, the confocal laser scanning microscopy (CLSM) images (Fig. 7D and E) revealed that MSN@BN1 displayed similar intracellular distribution and localization with BN1 in the 2D/3D assay. In general, bare MSN cannot effectively pass through the BBB (Fig. S27) [35]. While the transmission electron microscopy images (Fig. 7F) showed that the vesicle emerged from the apical side and moved downward after BBB models were incubated with MSN@BN1 for a different time, we again confirmed that BN1 as a vector could enhance the transport efficiency of MSN. The BN1-loaded MSN were then administered by tail vein injection in mice (*n* = 3). We then analyzed the biodistribution of MSN@BN1 (Fig. 7G). Small vesicles containing MSN was observed in the brain microvascular section, showing the successful BBB penetration. Besides, CLSM was also used to monitor MSN@BN1 transport efficiency after injection. The red fluorescence (Fig. S28) from BN1 revealed the most effective uptake by the vascular endothelial cell. Taken together, these results were in good agreement with our above free BN1 data and demonstrate the in vivo transport of MSN across the BBB using BN1-functionalized.

## Discussion

BBB presents one of the most substantial challenges for delivering therapeutic agents to the CNSs and, not surprisingly, is an important target for clinical neurology [45]. BBB is impermeable to many molecules by creating continuous tight junctions and overexpressing several types of molecular efflux pumps. Enhanced therapeutics entry to brain-cross BBB remains the most critical issue in various CNS diseases. However, current studies indicate that the high selectivity of the BBB still restricts the entry of all macromolecular drugs and over 98% of small-molecule drugs into the brain [15]. In this context, there is an urgent need to address these bottlenecks by developing novel drug delivery methods that can efficiently transport therapeutic agents across the BBB without compromising its normal structure and function.

The diffusion distribution of drugs in the extracellular space of the brain is regulated by various factors, including blood flow, cerebrospinal fluid dynamics, movement of extracellular fluid, pH value, presence of extracellular matrix, and other factors [46]. However, because of these regulatory mechanisms, an amount of drugs that can effectively reach the BBB site for transport is substantially reduced. Consequently, the efficiency of concentration-dependent free diffusion alone is lowly. To overcome this limitation, active transport mechanisms are used [47]. In this work, we synthesized and selected a red-emission pyran nitrile derivative varying their polarity to explore a new entry route to the brain in this work.

To conduct a systematic and efficient screening of drugs or target groups, several approaches have already been developed to model the BBB and study transport mechanisms across BMECs for investigating neurological pathways and evaluating the effectiveness of biopharmaceuticals and medicines [13,47]. The effective delivery and retention of drugs in tissues are crucial for successful treatment and reducing drug doses. Cell treated with BN1 displayed intense luminescence with cocultured 3D BBB model having the most intense signal from the cytoplasm, while the bEnd.3 cells from their membrane in 2D BBB model (Fig. 3A and B). It is known that the 3D BBB models as artificial frameworks could mediate the BBB flux. Coculture 3D BBB model always exhibited an improved polarized BBB phenotype induced by astrocytes [45]. Hence, it is not surprising to observe such phenomena since BBB 3D model (astrocyte/pericyte coculture) exhibited an improved BN1 distribution. Time-dependent assay demonstrated that BN1 was internalized through the endothelial cell layer efficiently, which could describe by kinetic model (Fig. 3E).

Molecules across the BBB are typically based on 4 potential routes: (a) active transport through receptor-mediated transcytosis, (b) molecular transporter targeting, (c) passive diffusion, and (d) carrier-mediated influx via solute carriers [11]. The transmembrane transport mechanism of hydrophilic molecules is commonly attributed to the expression of several highly specific SLCs within the BBB [48]. The individual knockout of classical SLC organic cation transporters resulted in an augmented uptake of BN1. These findings suggest that BN1 may utilize alternative pathways. Receptor-mediated transcytosis is a highly selective and noninvasive delivery approach. The BN1 compound exhibits prominent globular fluorescence signal in the 3D BBB model within a short duration (Figs. 3A and 4F). It is important to consider that the uptake of BN1 is associated with lysosomal or clathrin-mediated endocytosis (Fig. 4). Well-documented endocytosis and active transport inhibitors were used to investigate the cellular uptake mechanisms [49]. It is verified that a soluble compound BN1 with one quaternary ammonium salt group enables efficient penetration of the BBB via microtubule-mediated transcytosis (Fig. 4A). The ionic interaction between the amino cationic moiety of BN1 and the anionic component of the membrane, primarily the phosphate group in the phospholipid layer, induces local invagination of the cell membrane and facilitates BN1 absorption by the cell membrane. Subsequently, phospholipids reassemble to form an inverted microclump, releasing BN1 into the cytoplasm through a hydrophilic cavity formed by this microclump.

To systematically validate the feasibility of BN1 BBB permeation, in vivo imaging and ex vivo fluorescence staining assays were further conducted. With the assistance of molecular imaging techniques and the excellent optical properties, BN1 could penetrate the BBB efficiently in 3 min and remained at detectable levels even after 2 h for in vivo near-IR imaging (Fig. 5A). The fluorescence signal diminished slowly, which may be attributed to the fact that more BN1 was retained in brain parenchyma by crossing BBB (Fig. 5C). The in vivo imaging results were further confirmed by ex vivo fluorescence staining of frozen brain sections, which the fluorescent signal for BN1 was observed in pericytes located within the brain parenchyma, adjacent to the capillary endothelial cells (Fig. 6B).

The ability of hydrophilic small molecules to target the CNS without a drug carrier is intriguing. To demonstrate the compound efficient BBB penetration, BBB-impermeable MSN was functionalized loading with the compound, which could markedly enhance the transport efficiency as a codelivery system (Fig. 7D to G). The above findings demonstrate that aqueous molecules under ingenious design allow BBB penetration and can be used as a new delivery strategy for designing CNS targeting materials.

In summary, we successfully designed and characterized a small pyran nitrile polar derivative BN1 with quaternary ammonium salt group that can cross the BBB through endosome transcytosis. The systematic in vitro BBB model experiments indicated that BN1 could cross BBB freely via microtubule-mediated transcytosis. We have also demonstrated the successful delivery of BN1 into the CNS and penetrating the parenchyma in vivo. What is more, we prove further results both in vitro and in vivo and provide strong evidence, which confirmed that the novel aqueous compounds could be substantially enhanced by the presence of BN1-functionalized MSNs. The current research further suggests that BN1 could serve as the potential surface-functionalized group for imaging and treatment of brain diseases by hijacking microtubule-mediated transcytosis.

## Ethical Approval

All animal experiments were conducted according to the protocol approved by the Institutional Animal Care and Use Committee of West China Hospital of Sichuan University, registered as 2021-1475A.

## Data Availability

The datasets used and/or analyzed during the current study are available from the corresponding author on reasonable request
