# Supplementary material for: Advancing Central Nervous System Drug Delivery with Microtubule-Dependent Transcytosis of Novel Aqueous Compounds
Source: Biomater Res. 2024 Jul 24;28:0051. doi: 10.34133/bmr.0051 (PMC11268840; doi:10.34133/bmr.0051)
Supplement: Supplementary 1 — Figs. S1 to S28 Table S1 and S2 [file bmr.0051.f1.docx]

**†Supporting information**

**Advancing CNS Drug Delivery with Microtubule-Dependent Transcytosis of Novel Aqueous Compounds**

Content

[**Synthetic procedures** 3](#_Toc166187387)

[**Table S1.** Primer sequences for RT-PCR 5](#_Toc166187389)

[**Fig.** **S1** The plasmid profile of the vector. 6](#_Toc166187390)

**Fig. S2**. Schematic representation of the synthesis procedures of BN, BN1, BN2. 5

[**Fig. S3** HR-MS spectrum of BN 7](#_Toc166187391)

[**Fig. S4** ^1^H-NMR spectrum of BN in CDCl_3_. 7](#_Toc166187392)

[**Fig. S5** HR-MS spectrum of BN1 8](#_Toc166187393)

[**Fig. S6** ^1^H-NMR spectrum of BN1 in DMSO 8](#_Toc166187394)

[**Fig. S7** HR-MS spectrum of BN2 9](#_Toc166187395)

[**Fig. S8** ^1^H-NMR spectrum of BN2 in DMSO 9](#_Toc166187396)

[**Table S2.** Excitation energies, corresponding wavelengths, oscillator strengths and major contribution for BN, BN1, BN2. (H: HOMO, L: LUMO). 10](#_Toc166187397)

[**Fig. S9** UV-*vis* absorption a) and fluorescence emission b) spectra of BN, BN1 and BN2 in 10 μΜ DMSO solution. 10](#_Toc166187398)

[**Fig. S10** Representation of calculated HOMO and LUMO orbitals of BN, BN1 and BN2 11](#_Toc166187399)

[**Fig. S11** Two-photon absorption cross sections BN, BN1 and BN2 (50 mM) in DMSO solutions from 800 nm to 1060 nm 11](#_Toc166187400)

[**Fig. S12** Fluorescence decay of BN, BN1 and BN2 12](#_Toc166187401)

[**Fig. S13** HeLa and b.End3 cells stained with BN, BN1, BN2, respectively Scale bars 25 μm 12](#_Toc166187402)

[**Fig. S14** One-photon (green) and Two-photon (red) micrographs for living b.End3 cells treated with BN1 (5 μM) co-stained with DAPI and merged with DIC channel. Scale bar 20 μm 12](#_Toc166187403)

[**Fig. S15** HeLa cells treated with BN1 (5 Μm, 30 min) and co-stained cell mask green. Scale bar 20 μm 13](#_Toc166187404)

[**Fig. S16** The MTT assay using Hela and b.End3 cells for 24 hours treated with BN1 in the bioimaging concentration. 13](#_Toc166187405)

[**Fig. S17** Immunofluorescence of tight junction proteins a) (claudin-5 and ZO-1) (red) for 2D bEnd.3 monolayer and 3D BBB model. Scale bar: 20 μm. b) Transition of TEER after thawing until the start of the experiment. 13](#_Toc166187406)

[**Fig. S18** Depth code of 3D Z-stack transwell insert microporous membrane and Relative luminescence intensity of BN1 in cell media of lower compartments after treat with BN1 for different time. Scale bars 20 μm**.** 14](#_Toc166187407)

[**Fig. S19** a) 3D bEnd.3 monolayer on glass bottom dishes (collagen coated) treated with BN1 for different time. White dashed show the cell membrane. Scale bar 20 μm. b) Real time image of 3D model treatment with BN1. Scale bars 20 μm 14](#_Toc166187408)

[**Fig. S20** The effect of BN1 on the expression of organic cation transporters mRNAs in BBB model were measured by RT-PCR and normalised with β-actin. 15](#_Toc166187409)

[**Fig. S21** The confocal micrographs of BN1 after knockdown of the three organic cation transporters genes in bEnd.3 cells a) and the relative luminescence intensity in the cells b). 15](#_Toc166187410)

[**Fig. S22**. Flow cytometry intensity assay for cellular uptake of BN1 after cells treated with different temperature. 15](#_Toc166187411)

[**Fig. S23**. a) Z-stacks reconstructed into 3D images of 3D BBB model treated with low temperature. b) Fluorescence analysis the BN1 in cell media of lower compartments after different temperature treatment. Scale bars 25 μm. 15](#_Toc166187412)

[**Fig. S24** The 3D viewer of glomerulus a) and renal tubule b) confocal micrographs from mice after multiple *i.v.* injection of 0.1 mM of BN1 after 24 h. 16](#_Toc166187413)

[**Fig. S25** Confocal micrographs of brain sections from mice after *i.v.* injection of BN1, Immunofluorescence assays to show neurosynaptic (MAP2) neuronal (NeuN) and astrocytes (GFAP), respectively. Scale bars 100 μm 16](#_Toc166187414)

[**Fig. S26** The data on the size of MSN and MSN@BN1 17](#_Toc166187415)

[**Fig. S27** TEM images of 2D b.End3 treated with MSN and stained solely with osmium tetroxide. Scale bars 1μm. 17](#_Toc166187416)

[**Fig. S28** The micrographs showed the detail of brain capillaries and MSN@BN1 signal imaged by confocal laser scanning microscopy after i.v. injection of MSN@BN1. Scale bars 20 μm. 18](#_Toc166187417)

[**Notes and references** 18](#_Toc166187418)

**Synthetic procedures**

**Synthesis of OL, ON1 and ON2**: OL were prepared refering Li’s work [1]. ON1 and ON2 were prepared according to the similar procedures with our previous work excepted that compound I were replaced by 4-(ethyl(2-hydroxyethyl)amino)benzaldehyde and 4-(bis(2-hydroxyethyl)amino) benzaldehyde [2].

**Preparation of M3**: M2 (2.00 g, 12.50 mmol) and malononitrile (8.30 g, 125.0 mmol) were added to 15 mL acetic anhydride. The solution was refluxing with adding 20 mL H_2_O for 2 h after the above reflux reaction was proceed for 4 h. After cooling to room temperature, the brown organic phase was concentrated under reduced pressure. The product obtained was purified by silica gel chromatography column (V_petroleum ether_: V_ethylacetate_ = 15:1). Yield: 46 %.

**Preparation of BN:** M3 (1.18 g, 5.67 mmol) and OL (1.00 g, 5.67 mmol) was dissolved in acetonitrile (20.0 mL). With constant stirring, five drops of piperidine were added to the mixture, and the solution was refluxed for 12 hours to produce a red solution. The solution was concentrated under reduced pressure to yield red oil, which was purified by column chromatography using dichloromethane/ methanol (10:1, v/v) as the eluent, and BN was collected as red solid (0.51 g, 28 %). C_24_H_21_N_3_O, Calcd (%): C, 78.45; H, 5.76; N, 11.44. Found: C, 78.30; H, 5.77; N, 11.35. ^1^H-NMR (CDCl_3_, 400 MHz, ppm) δ = 8.89 (d, J = 8.3 Hz, 1 H), 7.69 (t, J = 7.8 Hz, 1 H), 7.53 (t, J = 11.2 Hz, 2 H),7.47 – 7.35 (m, 3 H),6.75 (s, 1 H), 6.67 (d, J = 8.2 Hz, 2 H), 6.52 (d, J = 15.7 Hz, 1 H),3.44 (q, J = 7.0 Hz, 4 H), 1.22 (t, J = 7.1 Hz, 6 H). ^13^C-NMR (*d_6_* -DMSO, 100 MHz, ppm) δ = 159.70, 152.45, 152.03, 149.46, 140.27, 134.92, 130.67, 125.81, 124.50, 121.59, 118.83, 117.19, 112.45, 111.36, 104.69, 43.83, 12.48. FT-IR (KBr, ν, cm^-1^ ): 2966 (w), 2201 (s), 2186 (m), 1588 (s), 1550 (vs), 1496 (m), 1477 (s), 1456 (m), 1438 (m), 1401 (s), 1364 (w), 1341 (w), 1327 (w), 1264 (s), 1228 (w), 1208 (m), 1182 (s), 1152 (m), 1143 (w), 1075 (w), 1011 (w), 978 (m), 963 (m), 865 (w), 833 (w), 807 (m), 766 (m), 748 (m), 709 (w). HRMS-ESI: m/z, cal: 368.18, found: 368.18 [M + H]^+^. M.p. = 178 - 181 °C.

The BN1 and BN2 were synthesized according to the similar procedures.

**Preparation of BN1**: C_27_H_30_F_6_N_4_OP, Calcd (%): C, 56.84; H, 5.12; N, 9.82. Found: C, 56.30; H, 5.17; N, 9.95. 1H-NMR (*d_6_* -DMSO, 400 MHz, ppm) δ = 8.73 (d, J = 8.4 Hz, 1 H), 7.90 (d, J =7.8 Hz, 1 H), 7.83 – 7.55 (m, 5 H), 7.23 (d, J = 15.8 Hz, 1 H), 6.93 (s, 1 H), 6.83 (d, J =8.7 Hz, 2 H), 3.83 (d, J = 8.5 Hz, 2 H), 3.61 – 3.44 (m, 4 H), 3.18 (s, 9 H), 3.12 – 3.06 (m,1 H), 1.16 (t, J = 6.8 Hz, 3 H). ^13^C-NMR (*d_6_* -DMSO, 100 MHz, ppm) δ = 181.94, 163.89, 159.56, 152.88, 151.75, 148.88, 139.75, 135.14, 130.51, 125.97, 124.68, 119.53, 116.03, 114.28, 112.00, 104.66, 57.86, 52.51, 42.84. FT-IR (KBr, ν, cm^-1^ ):2205 (w), 1592 (s), 1554 (s), 1520 (m), 1498 (m), 1481 (s), 1454 (m), 1434 (w), 1406 (s),1398 (m), 1357 (w), 1264 (m), 1209 (w), 1185 (s), 1157 (w), 1145 (w), 978 (m), 915 (w), 858 (w), 839 (s), 807 (w), 770 (w), 558 (m). HRMS-ESI: m/z, cal: 425.23, found: 425.23 [M - PF_6_^-^]^+^. M.p. = 303-306 °C.

**Preparation of BN2:** C_30_H_37_F_12_N_5_OP_2_, Calcd (%): C, 46.58; H, 4.82; N, 9.05. Found: C, 46.40; H, 4.87; N, 9.75. ^1^H-NMR (*d_6_* -DMSO, 400 MHz, ppm) δ = 8.72 (d, J = 8.3 Hz, 1 H), 7.90 (d, J = 7.6 Hz, 1 H), 7.87– 7.64 (m, 4 H), 7.60 (d, J = 7.7 Hz, 1 H), 7.28 (d, J = 15.9 Hz, 1 H), 6.93 (d, J = 10.0 Hz,3 H), 3.89 (s, 4 H), 3.53 (s, 4 H), 3.20 (s, 18 H). ^13^C-NMR (*d_6_* -DMSO, 100 MHz, ppm) δ = 159.04, 152.74, 152.01, 148.06, 139.13, 135.19, 130.40, 125.99, 124.60, 118.92,117.48, 117.10, 116.19, 115.09, 112.83, 105.53, 60.52, 58.60, 52.69, 43.17.FT-IR (KBr, ν, cm^-1^ ): 2210 (s), 1628 (s), 1554 (s), 1522 (m), 1500 (m), 1481 (s), 1455 (m), 1401 (s), 1337 (m), 1309 (w), 1264 (s), 1209 (w), 1184 (s), 1154 (w), 1142 (w), 979 (m),921 (m), 840 (s), 768 (w), 743 (w), 557 (m). HRMS-ESI: m/z, cal: 241.65, found: 241.65 [M - 2PF_6_^-^]^2+^. M.p. = 290-293 °C.





**Table S1.** Primer sequences for RT-PCR

| Target  genes | Forward primer | Reverse primer |
| --- | --- | --- |
| SLC22A2 | CCAGTGCATGAGGTATGAGGT | CTGAAACAGGTCCAGCATCCA |
| SLC22A5 | ACTGTGCCAGGGGTGCTAT | GCAACTGAGGCTTCGTAGAAT |
| SLC47A1 | GTTGGCCTTACGGAGAGGAC | AATCCCACCCACCAAGACTAA |
| β-actin | GGCTGTATTCCCCTCCATCG | CCAGTTGGTAACAATGCCATGT |


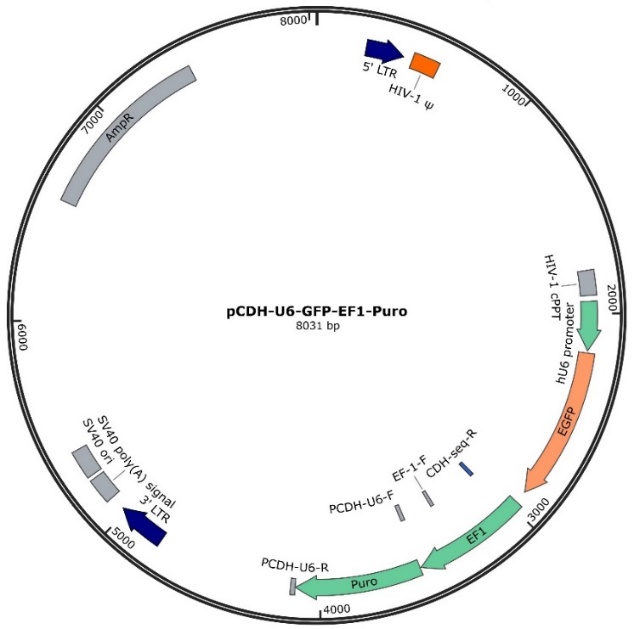


**Fig.** **S1** The plasmid profile of the vector.


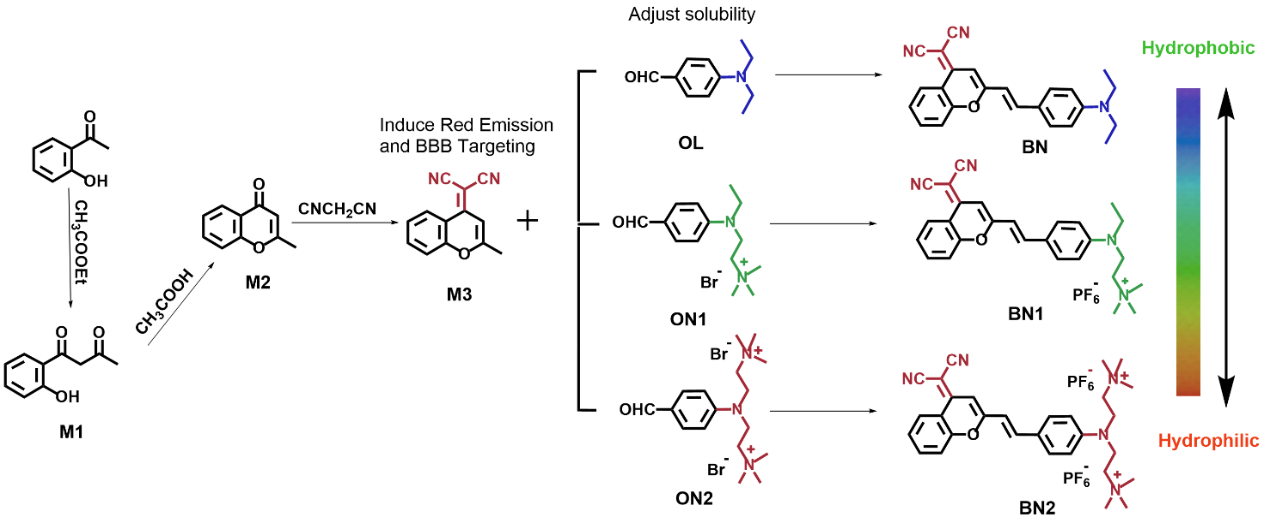


**Fig. S2** Schematic representation of the synthesis procedures of BN, BN1, BN2.


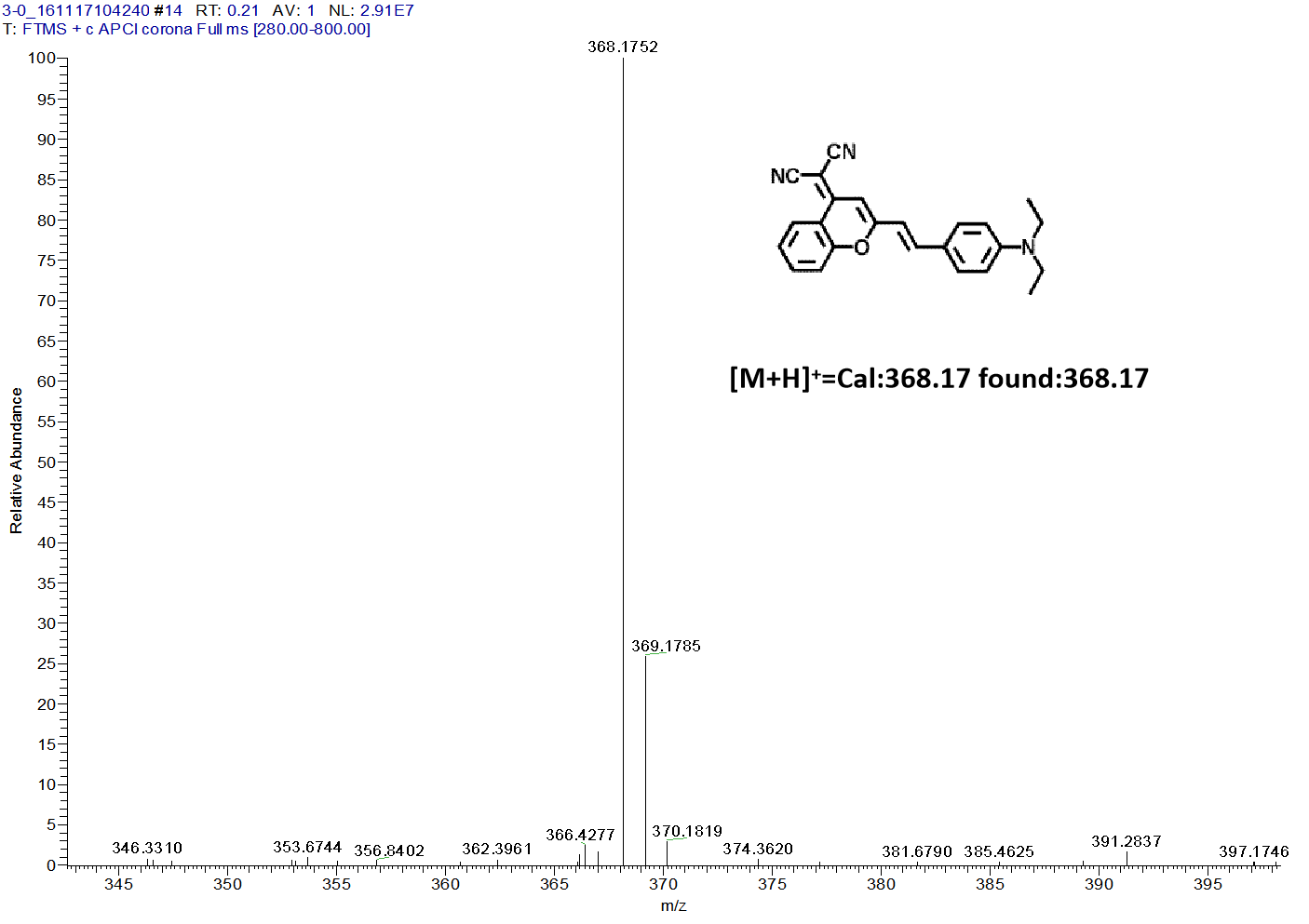


**Fig. S3** HR-MS spectrum of BN


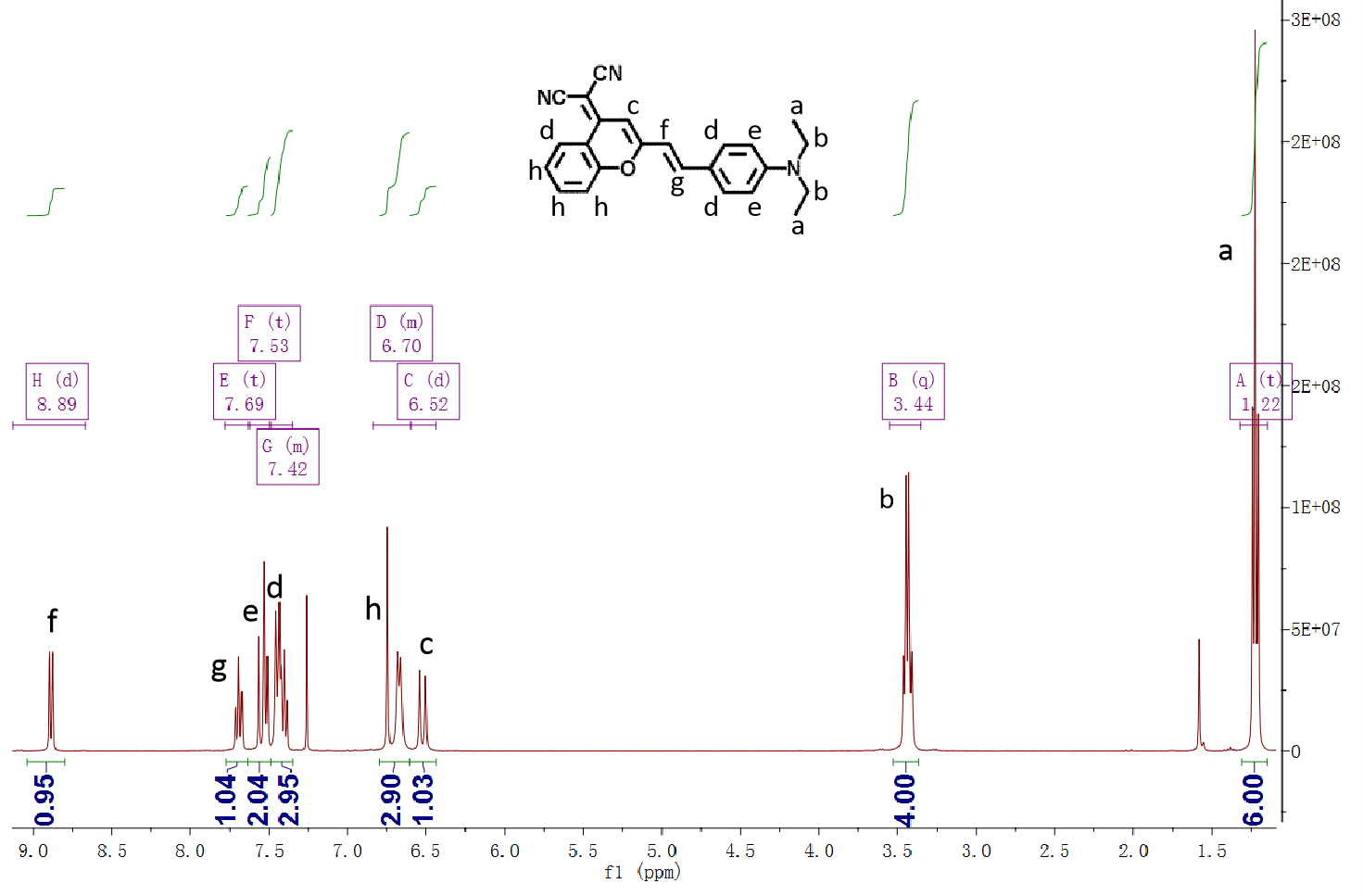


**Fig. S4** ^1^H-NMR spectrum of BN in CDCl_3_.


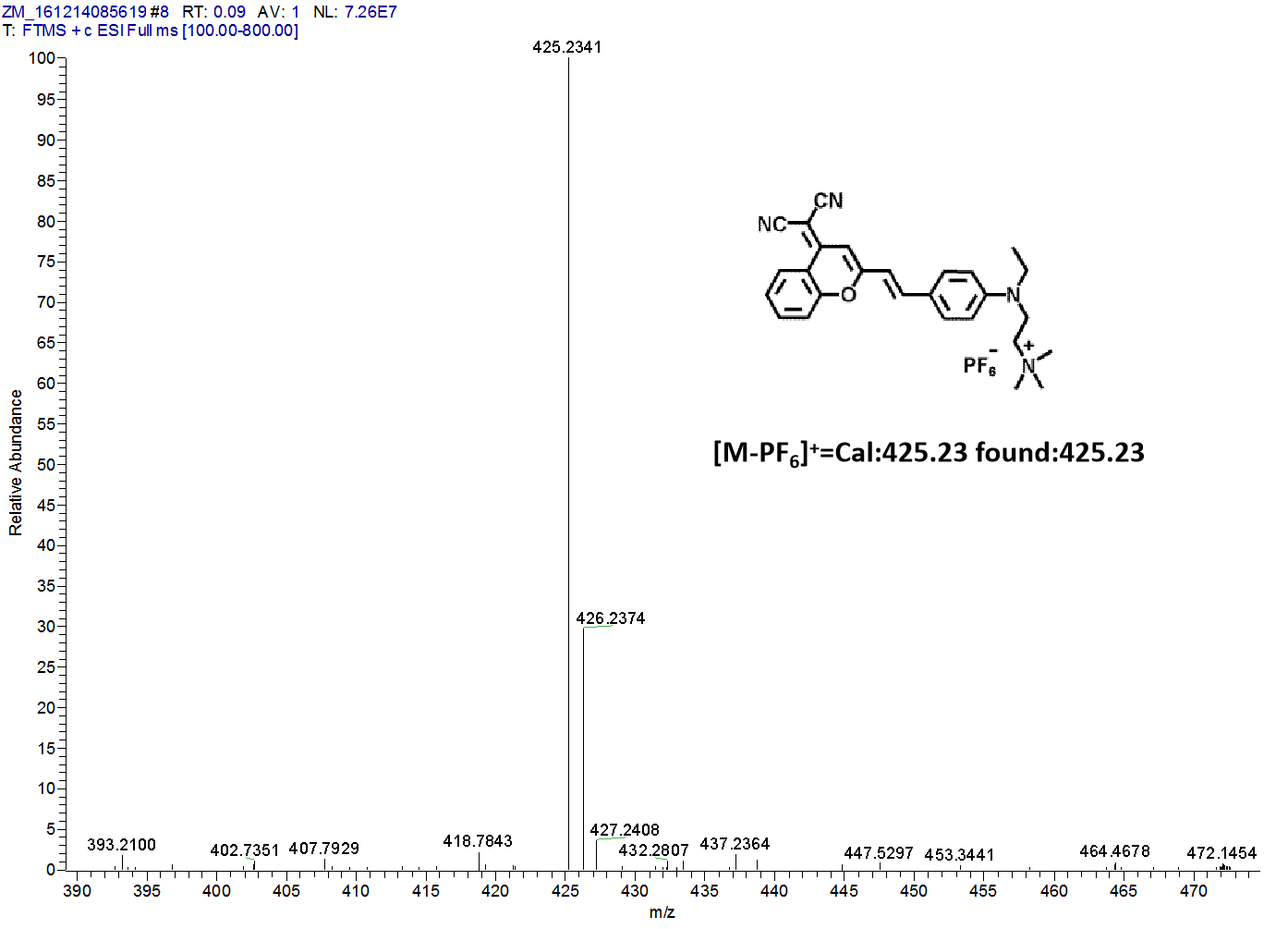


**Fig. S5** HR-MS spectrum of BN1


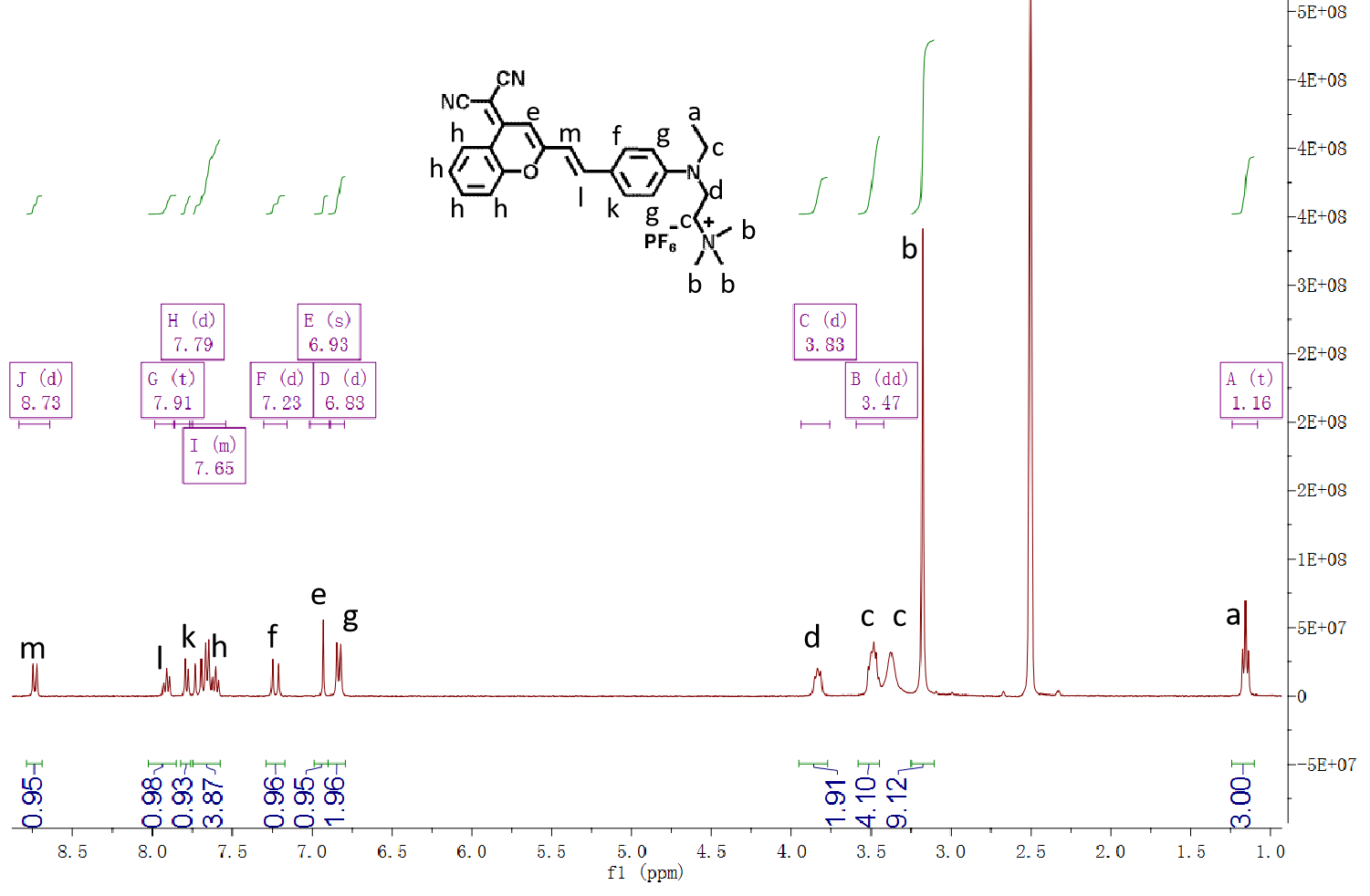


**Fig. S6** ^1^H-NMR spectrum of BN1 in DMSO


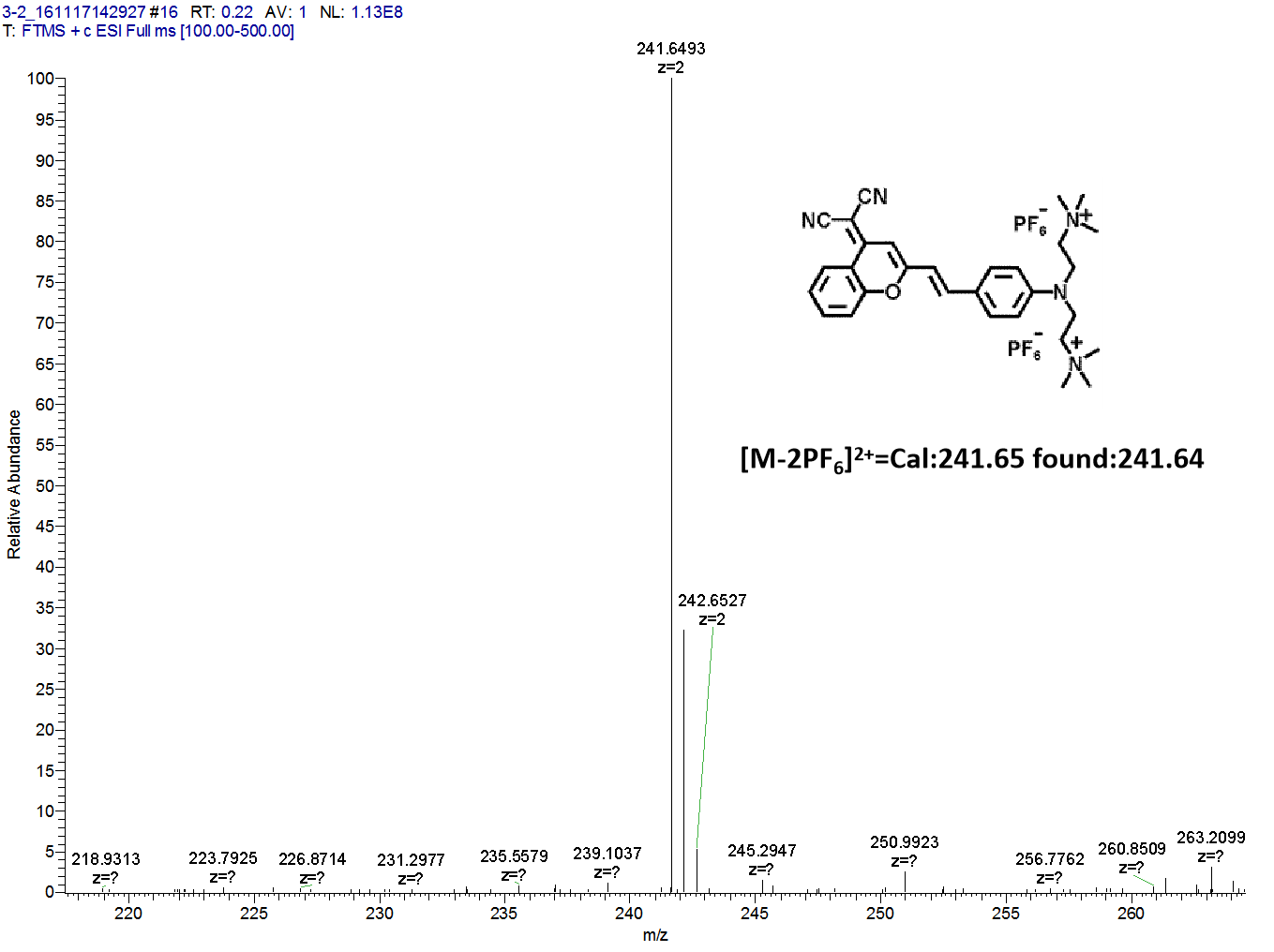


**Fig. S7** HR-MS spectrum of BN2


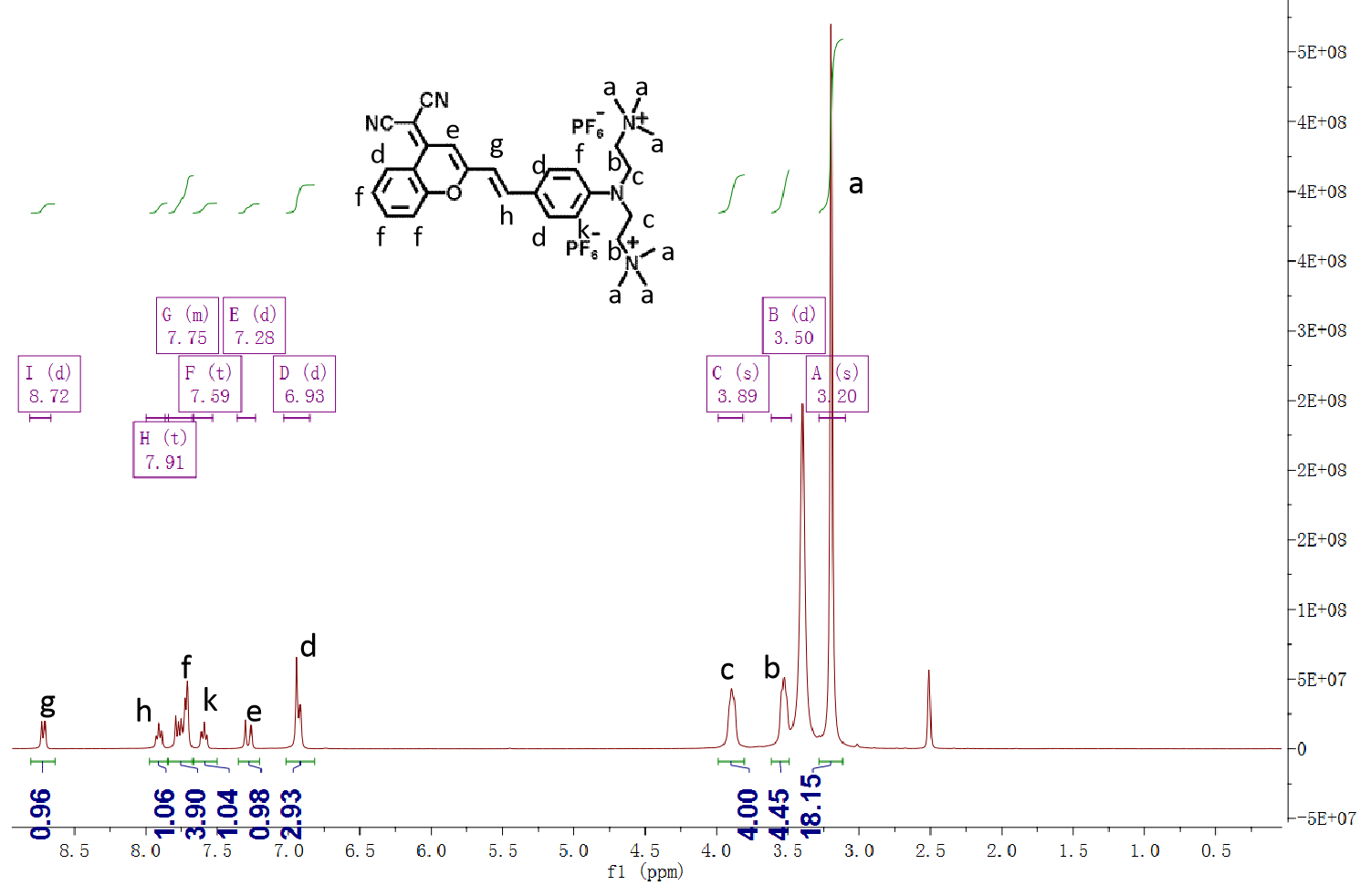


**Fig. S8** ^1^H-NMR spectrum of BN2 in DMSO

**Table S2.** Excitation energies, corresponding wavelengths, oscillator strengths and major contribution for BN, BN1, BN2. (H: HOMO, L: LUMO).

| Compound | ΔE(eV)^[a]^ | λ (nm)^[b]^ | λ (nm)^[c]^ | Oscillator strengths | Nature of the transition |
| --- | --- | --- | --- | --- | --- |
| BN | 2.7425 | 545.34 | 548 | 0.5837 | 97 (H)→99 (L+1) (0.67) |
| BN1 | 2.7167 | 516.37 | 520 | 0.6537 | 130 (H-2)→135 (L+2) (0.65) |
| BN2 | 2.5198 | 492.04 | 496 | 0.7733 | 112 (H-1)→114 (L) (0.73) |

[a] The energy gap of the single-photon absorption band. [b] peak position of the linear absorption band. [c] Experimental peak position of the linear absorption band.


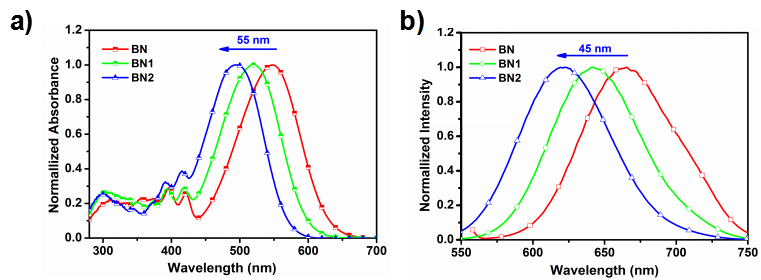


**Fig. S9** UV-*vis* absorption a) and fluorescence emission b) spectra of BN, BN1 and BN2 in 10 μΜ DMSO solution.

**
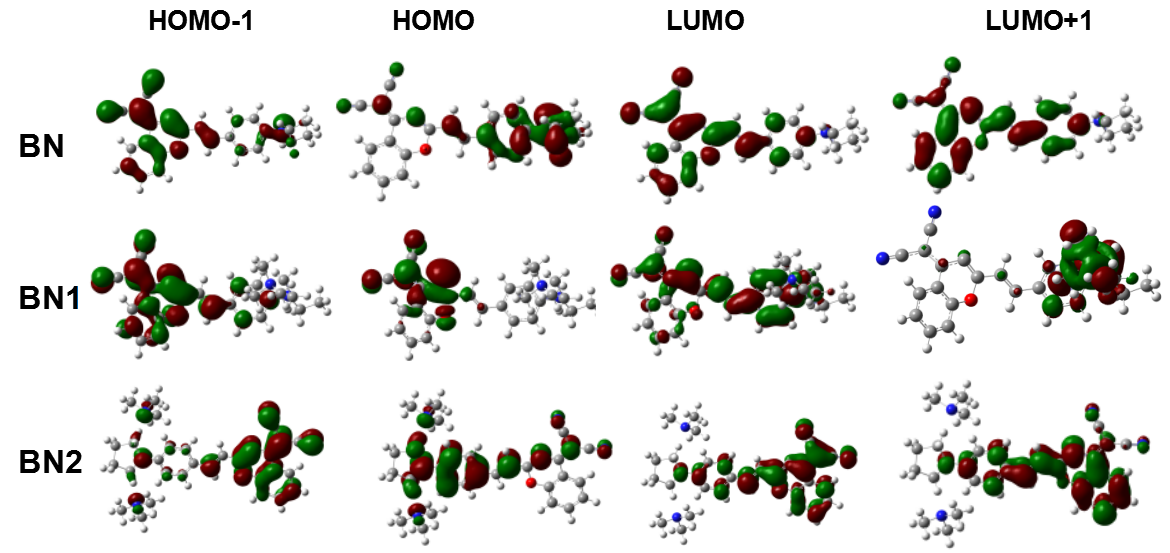
**

**Fig. S10** Representation of calculated HOMO and LUMO orbitals of BN, BN1 and BN2


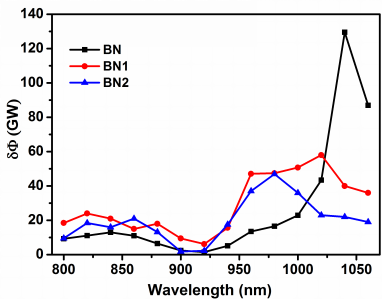


**Fig. S11** Two-photon absorption cross sections BN, BN1 and BN2 (50 mM) in DMSO solutions from 800 nm to 1060 nm


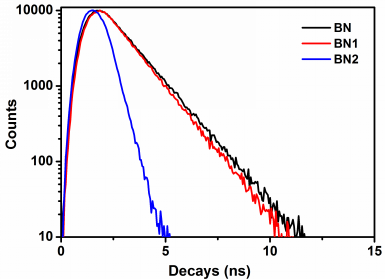


**Fig. S12** Fluorescence decay of BN, BN1 and BN2


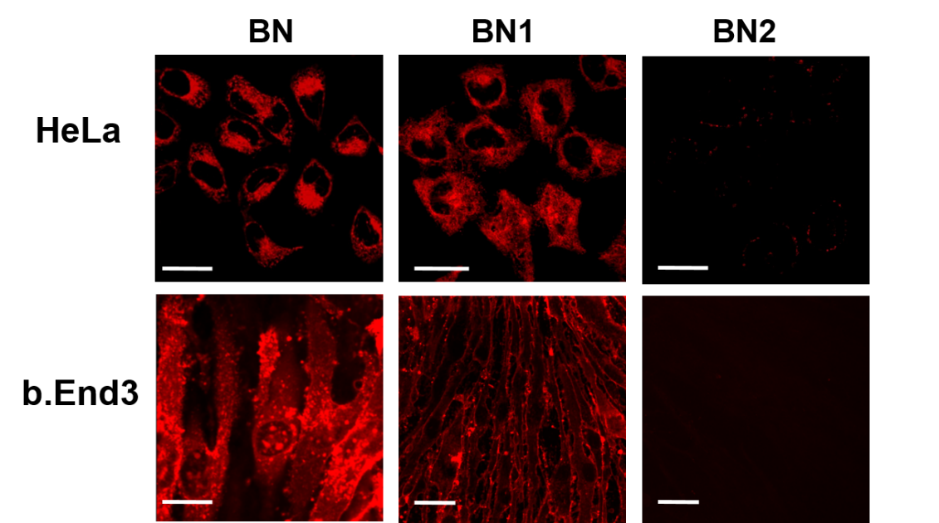


**Fig. S13** HeLa and b.End3 cells stained with BN, BN1, BN2, respectively Scale bars 25 μm


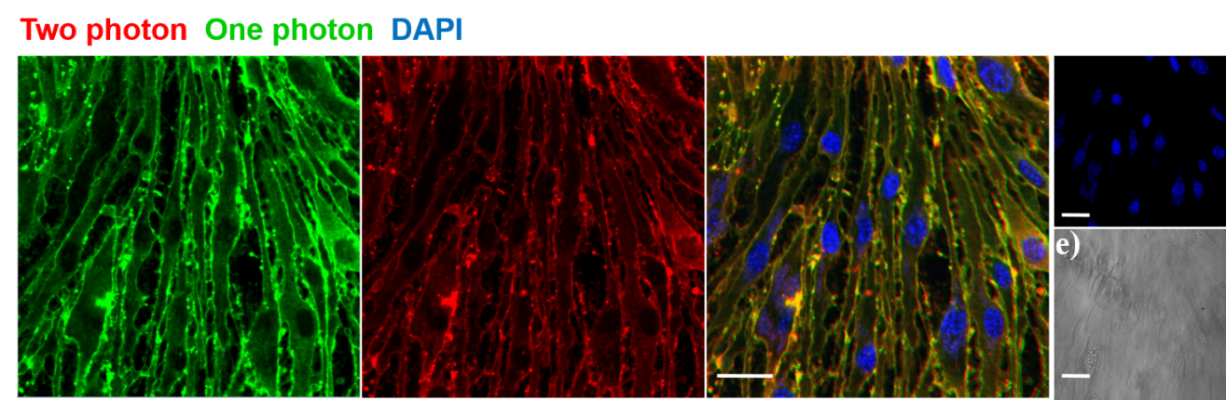


**Fig. S14** One-photon (green) and Two-photon (red) micrographs for living b.End3 cells treated with BN1 (5 μM) co-stained with DAPI and merged with DIC channel. Scale bar 20 μm


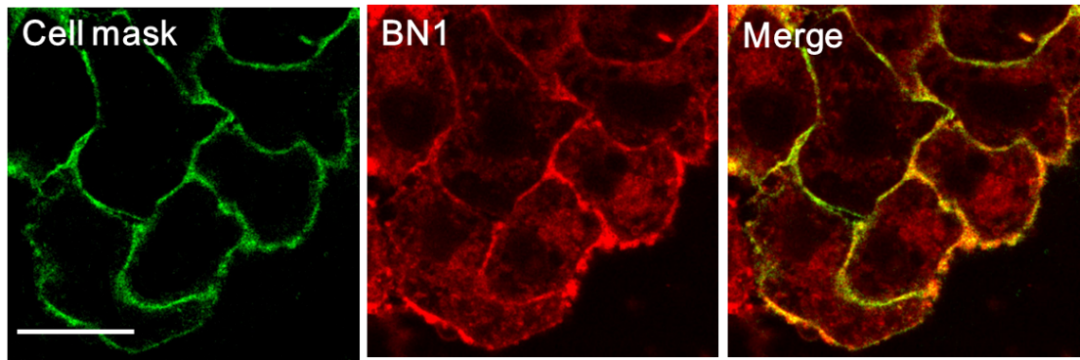


**Fig. S15** HeLa cells treated with BN1 (5 Μm, 30 min) and co-stained cell mask green. Scale bar 20 μm


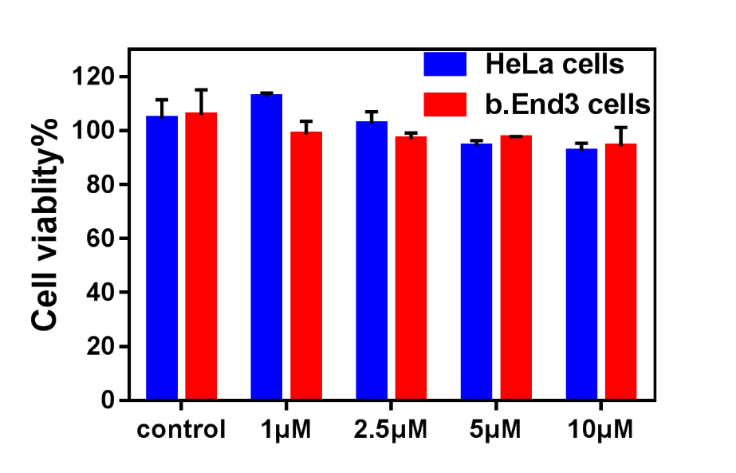


**Fig. S16** The MTT assay using Hela and b.End3 cells for 24 hours treated with BN1 in the bioimaging concentration.


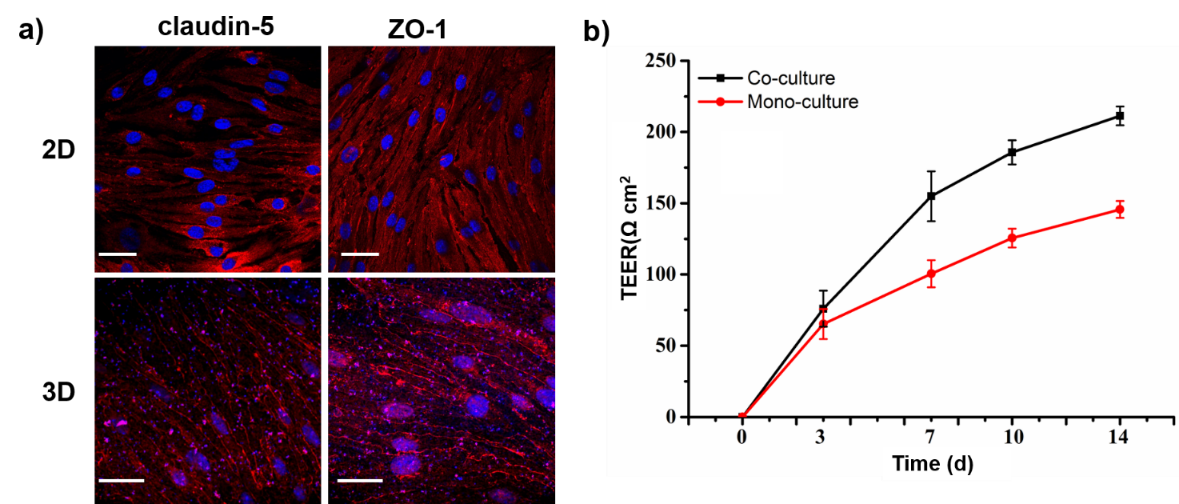


**Fig. S17** Immunofluorescence of tight junction proteins a) (claudin-5 and ZO-1) (red) for 2D bEnd.3 monolayer and 3D BBB model. Scale bar: 20 μm. b) Transition of TEER after thawing until the start of the experiment.


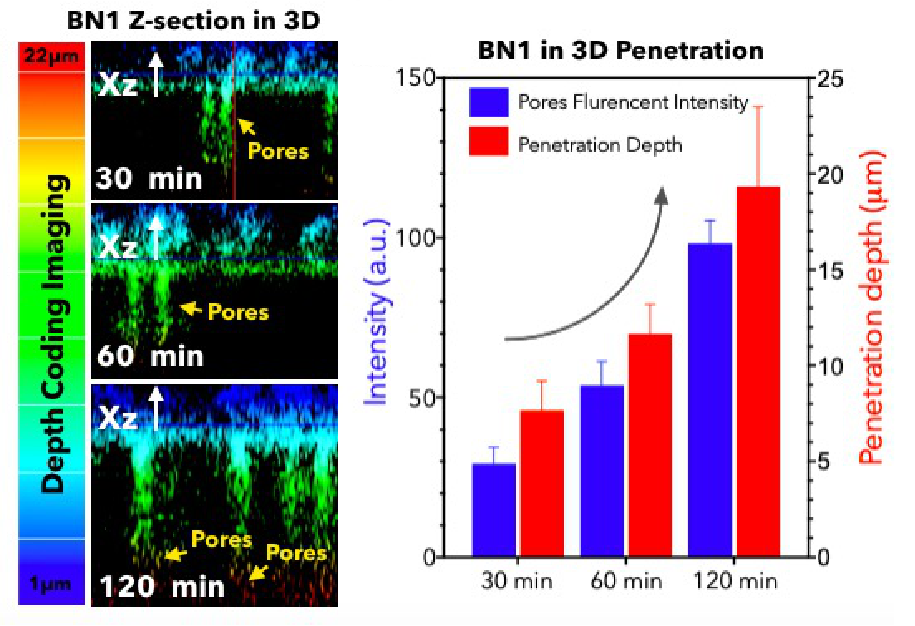


**Fig. S18** Depth code of 3D Z-stack transwell insert microporous membrane and Relative luminescence intensity of BN1 in cell media of lower compartments after treat with BN1 for different time. Scale bars 20 μm**.**


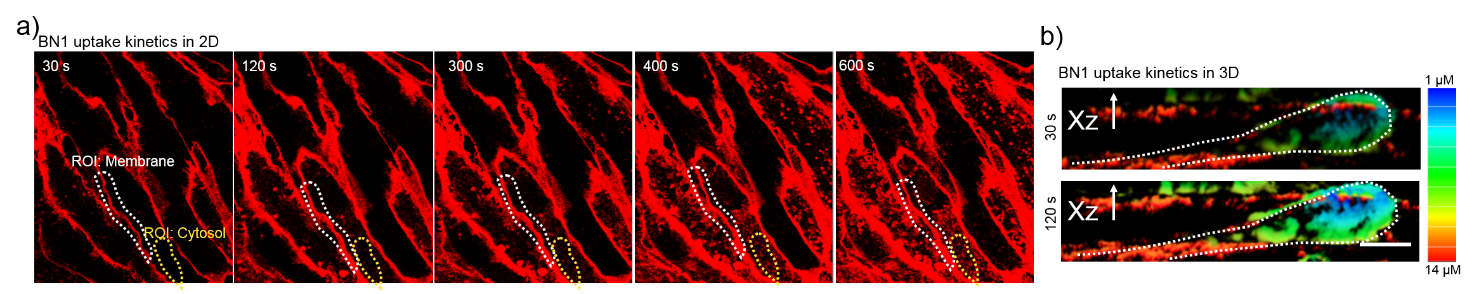


**Fig. S19** a) 3D bEnd.3 monolayer on glass bottom dishes (collagen coated) treated with BN1 for different time. White dashed show the cell membrane. Scale bar 20 μm. b) Real time image of 3D model treatment with BN1. Scale bars 20 μm


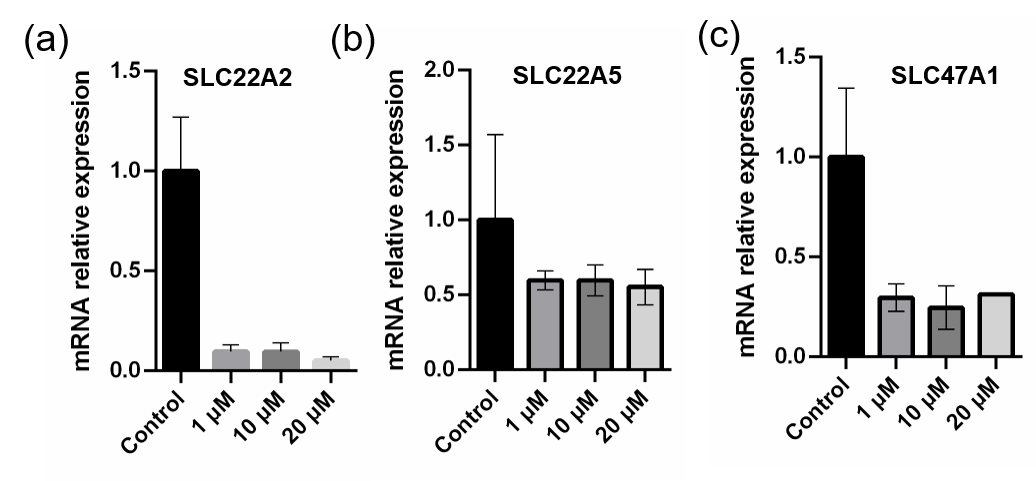


**Fig. S20** The effect of BN1 on the expression of organic cation transporters mRNAs in BBB model were measured by RT-PCR and normalised with β-actin.


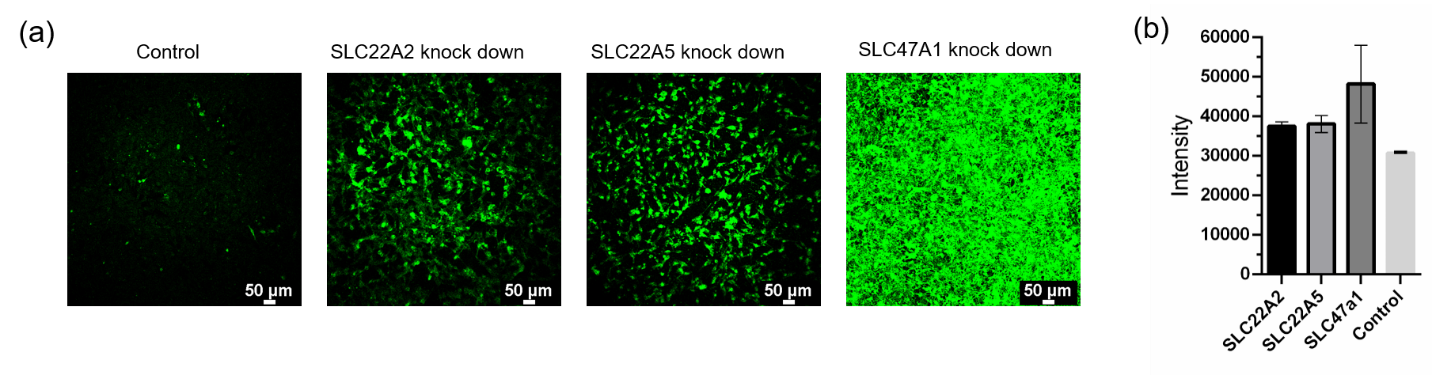


**Fig. S21** The confocal micrographs of BN1 after knockdown of the three organic cation transporters genes in bEnd.3 cells a) and the relative luminescence intensity in the cells b).


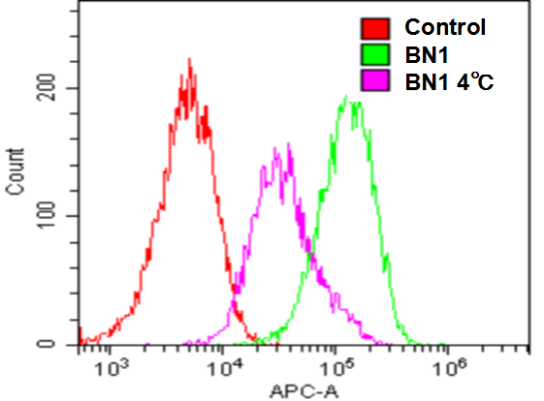


**Fig. S22**. Flow cytometry intensity assay for cellular uptake of BN1 after cells treated with different temperature.


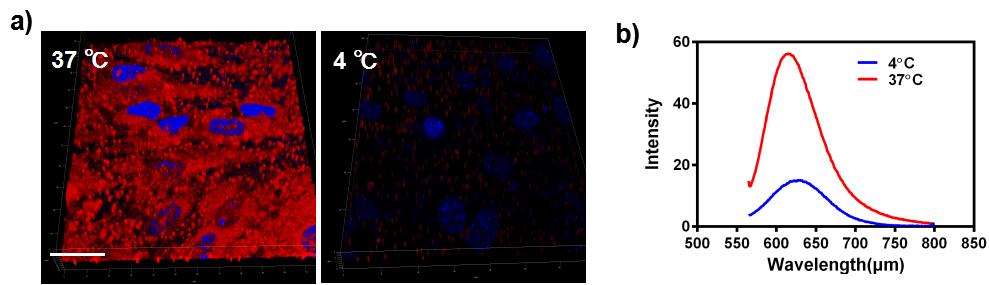


**Fig. S23**. a) Z-stacks reconstructed into 3D images of 3D BBB model treated with low temperature. b) Fluorescence analysis the BN1 in cell media of lower compartments after different temperature treatment. Scale bars 25 μm.


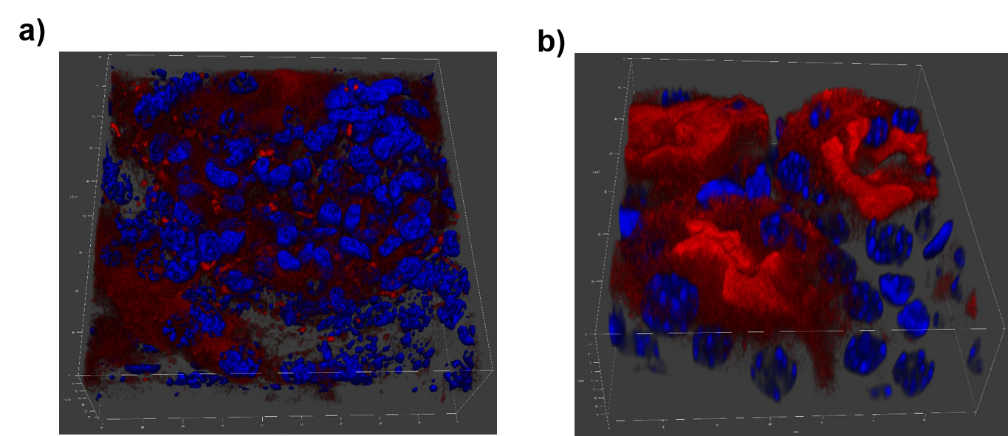


**Fig. S24** The 3D viewer of glomerulus a) and renal tubule b) confocal micrographs from mice after multiple *i.v.* injection of 0.1 mM of BN1 after 24 h.


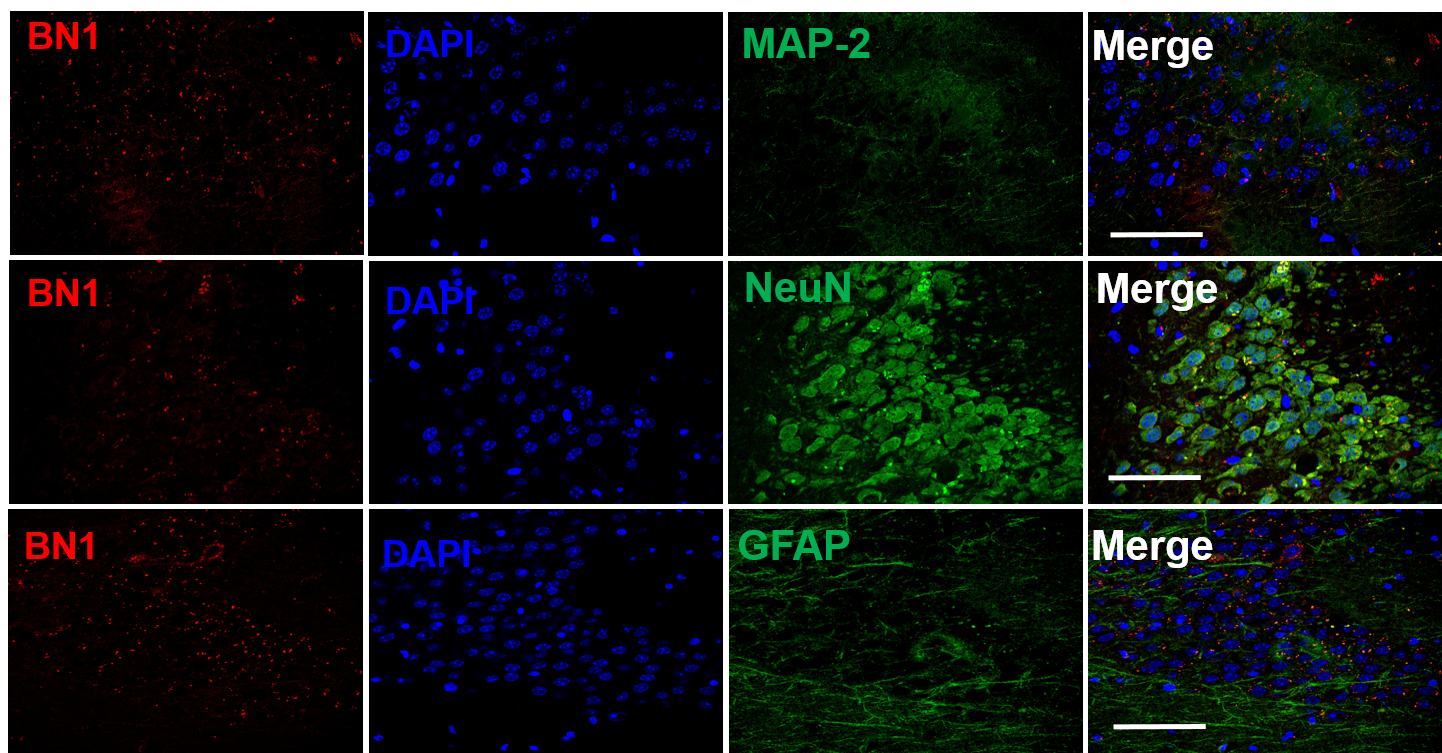


**Fig. S25** Confocal micrographs of brain sections from mice after *i.v.* injection of BN1, Immunofluorescence assays to show neurosynaptic (MAP2) neuronal (NeuN) and astrocytes (GFAP), respectively. Scale bars 100 μm





**Fig. S26** The data on the size of MSN and MSN@BN1


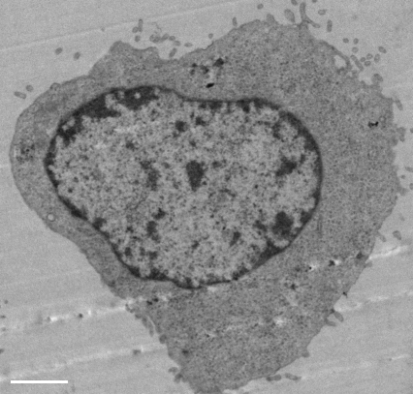


**Fig. S27** TEM images of 2D b.End3 treated with MSN and stained solely with osmium tetroxide. Scale bars 1μm.


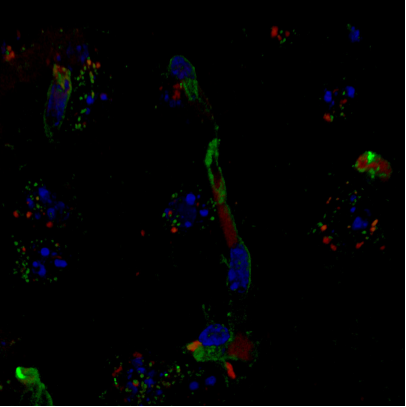


**Fig. S28** The micrographs showed the detail of brain capillaries and MSN@BN1 signal imaged by confocal laser scanning microscopy after i.v. injection of MSN@BN1. Scale bars 20 μm.

**Notes and references**

**1.** Li R, Li D, Fei W, et al. Synthesis, prodigious two-photon absorption cross sections and electrochemical properties of a series of triphenylamine-based chromophores. Optical Materials. 2014;36(8):1281-1288.

**2.** Hu L, Wang H, Xu X, et al. A small-molecule with large two-photon action cross-section serves as the membrane-permeable probe for live cells imaging and bacteria viability. Sensors and Actuators B: Chemical. 2017;241:1082-1089.
